# A Vibrational Analysis of Pyridoxal 5′-Phosphate Derivatives: Pyridoxal 5′-Phosphate-isopropylamine and Pyridoxal 5′-Phosphate‑(*S*)‑1-phenylethylamine

**DOI:** 10.1021/acs.jpcb.5c04584

**Published:** 2025-12-29

**Authors:** Ramandeep S. Dosanjh, Stewart F. Parker, Paul Collier, Ahir Pushpanath, Andrew P. E. York, Damian Grainger, Sanita B. Tailor, Timothy Johnson, Timothy Hyde, Lachlan J. N. Waddell, Andrew Sutherland, David Lennon

**Affiliations:** † School of Chemistry, Joseph Black Building, 3526 University of Glasgow, Glasgow G12 8QQ, U.K.; ‡ ISIS Facility, Rutherford Appleton Laboratory Harwell Campus, Chilton, Didcot OX11 0QX, U.K.; § 41975 Johnson Matthey Technology Centre, Blounts Court Road, Sonning Common, Reading RG4 9NH, U.K.; ∥ 5292 Johnson Matthey, 28 Cambridge Science Park, Milton Road, Cambridge CB4 0FP, U.K.

## Abstract

The external aldimine, a pivotal Schiff base intermediate in pyridoxal 5′-phosphate (PLP)-dependent enzyme-catalyzed reactions, plays a central mechanistic role in the ω-transamination pathway used for the synthesis of chiral amines in pharmaceutical production. To investigate the potential of vibrational spectroscopy to probe molecular interactions relevant to PLP’s role as a cofactor in transamination reactions, two external aldimines, pyridoxal 5′-phosphate-isopropylamine (PLP-IPAm) and pyridoxal 5′-phosphate-(*S*)-1-phenylethylamine (PLP-PEA), are synthesized and analyzed by vibrational spectroscopy. Single-molecule DFT calculations are employed to predict the vibrational characteristics of both compounds. Inelastic neutron scattering measurements validate the single-molecule DFT calculations for the noncrystalline PLP-aldimines. The computational data sets guide the assignment of ATR-IR and FT-Raman spectra of both external aldimines over the 400–4000 cm^–1^ range, enabling the identification of vibrational bands from functional groups that may contribute to the transamination mechanism. The characterization of discrete vibrational modes for each external aldimine defines a platform by which vibrational spectroscopy (e.g., Raman spectroscopy) could potentially be used to monitor PLP-dependent transamination processes.

## Graphical abstract

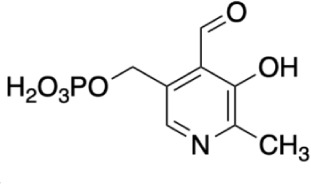

## 1 Introduction

Chiral amines represent a fundamentally important class of compounds across a wide range of chemical disciplines.^1^ In pharmaceutical development, they are particularly prominent, with approximately 40% of marketed drugs incorporating a chiral amine moiety.^2^ Beyond their critical role in medicinal chemistry, chiral amines also serve as key intermediates in the synthesis of complex natural products, agrochemicals, and functional materials. Their structural complexity and inherent ability to participate in hydrogen bonding confer valuable pharmacophoric properties and enable precise molecular recognition in both biological and synthetic systems.^3,4^ Given their widespread utility, significant efforts have been devoted to developing efficient and sustainable methods for their preparation. Among these, biocatalytic approaches have emerged as powerful tools, offering high levels of regio-, chemo-, and stereoselectivity, along with improved atom economy and environmental compatibility. Such strategies are increasingly central to modern synthetic chemistry, facilitating the streamlined production of optically active amines for diverse applications in science and industry.^5^

Within this framework, the current repertoire of biocatalysts for chiral amine production encompasses lipases,^6,7^ amine oxidases,^8,9^ imine reductases,^10^ and amine dehydrogenases.^11^ Among these, the utilization of transaminases (TAms) has emerged as an important strategy for chiral amine synthesis. This is due to distinct advantages that TAms offer, including high selectivity, enantioselectivity, and stability, which facilitate the direct transfer of an amine group from an amine donor to a ketone or aldehyde.^12,13^ Transaminases are broadly classified as α- or ω-transaminases, depending on the position of the amino group relative to the substrate’s carboxyl group.^2^ α-Transaminases act on substrates with an α-carboxyl group to form α-amino acids, while ω-transaminases can transfer a wide range of primary amino groups, to various keto acids, aldehydes, and ketones. The versatility and efficiency of ω-transaminases (ω-TAms) underscore their growing importance in industrial biocatalysis.^13,14^ ω-TAms have been widely employed in the preparation of pharmaceutical intermediates and bioactive compounds, through both kinetic resolution,^15–17^ or more commonly, direct asymmetric synthesis.^18,19^ A high-profile example is the ω-TAm driven synthesis of sitagliptin, a widely prescribed antidiabetic drug.^20^

Biocatalytic transamination reactions involving ω-TAms require the vitamin B6 derivative pyridoxal 5′-phosphate (PLP) as a crucial cofactor, a nonproteinaceous chemical species that enters the enzyme’s active site and provides catalytic functionality.^22,23^ PLP facilitates the transfer of functional groups between intermediary reaction substrates during transamination. The infrared and Raman spectra of PLP are known and signify the possibilities of hydrogen bonding interactions with the cofactor.^24,25^ While a comprehensive mechanistic understanding of ω-transaminases remains incomplete, it is generally assumed that the mechanism follows a cyclic process similar to the well-characterized mechanism observed in α-TAms.^21,26–28^ With reference to Scheme 1, the catalytic cycle begins with the formation of the internal aldimine via reaction of PLP with the ε-amino group of an active-site lysine. This is followed by transamination with the amino donor to form the external aldimine (*i*), C_α_ proton abstraction and formation of the quinonoid intermediate (*ii*), reprotonation at C_4_’ to yield the ketimine (*iii*), and hydrolysis to give the α-keto acid product and pyridoxamine phosphate (PMP) (*iv*). PLP is regenerated by reaction of PMP with a second keto acid substrate, completing the cycle.^23–26^ Among the intermediates formed during the transamination cycle, the external aldimine is a key structural and catalytic determinant that underpins the efficiency and specificity of PLP-dependent enzymes.^28^ This intermediate serves as a critical bridge between substrate binding and subsequent enzymatic transformations, effectively priming the active site for progression through the catalytic cycle.

**Scheme 1:**
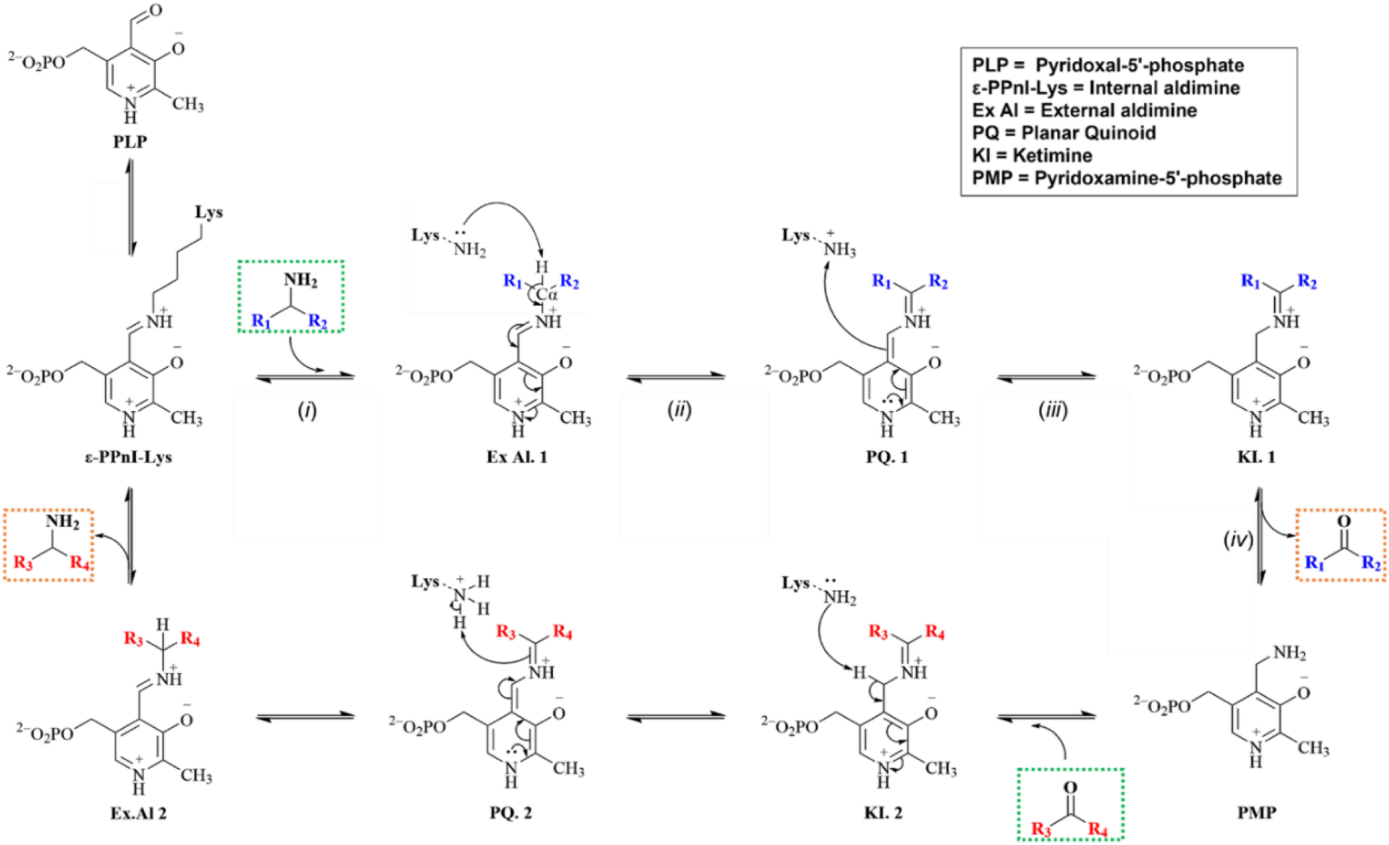
Generalized Mechanism of PLP-Assisted Transamination[Fn sch1-fn1]

i Adapted with permission from Cassimjee et al.^21^

The external aldimine passes through high-energy, rate-limiting transition states during its formation and catalytic transformation.^29^ Owing to its critical function in the PLP-dependent catalytic cycle, it has been extensively studied through both experimental and computational approaches. DFT-based investigations have proposed several mechanistic pathways for its formation via geminal diamine intermediates.^30,31^ Likewise, UV–Vis spectroscopy and hybrid quantum mechanical–molecular mechanical (QM/MM) studies have been employed to investigate stabilizing features, particularly the electron-sink effect of the PLP pyridine ring and the enolimine–ketoamine tautomeric equilibrium.^32–34^

The structural complexity of the external aldimine, which is fundamental to its catalytic function, gives rise to distinct vibrational signatures that can be sensitively probed using infrared (IR) and Raman spectroscopy. These nondestructive, noninvasive techniques are well suited to the characterization of reactive intermediates and labile functionalities.^35–38^ Nevertheless, direct *in situ* spectroscopic interrogation of the external aldimine within an enzyme active site remains challenging due to overlapping vibrational bands, protein background signals, and the structural heterogeneity and dynamic nature of the catalytic environment.^39^ An additional barrier is a limited understanding of the complexity inherent in the vibrational spectra of external aldimines, with complete vibrational assignments rarely available.

Against this background, to advance the option of using vibrational spectroscopy to probe the chemistry and interactions of PLP–amine Schiff bases, this study outlines a complete vibrational analysis of two well-defined model systems: pyridoxal 5′-phosphate isopropylamine (PLP-IPAm) and pyridoxal 5′-phosphate (*S*)-1-phenylethylamine (PLP-PEA) (Scheme 2). These compounds serve as chemically tractable analogues of external aldimine intermediates central to ω-transaminase catalysis. The choice of isopropylamine (IPAm) and (*S*)-1-phenylethylamine (PEA) is strategic: IPAm is widely utilized in biocatalysis due to its low cost, high reactivity, and facile removal of the byproduct, making it a benchmark substrate in transaminase studies.^40^ In contrast, PEA introduces aromaticity and stereocenters, enabling the investigation of electronic and steric effects on vibrational response and hydrogen-bonding interactions in the Schiff base motif. Together, these systems provide a foundation for correlating structural perturbations of PLP cofactor species with spectroscopic observables.^41–43^

**Scheme 2:**
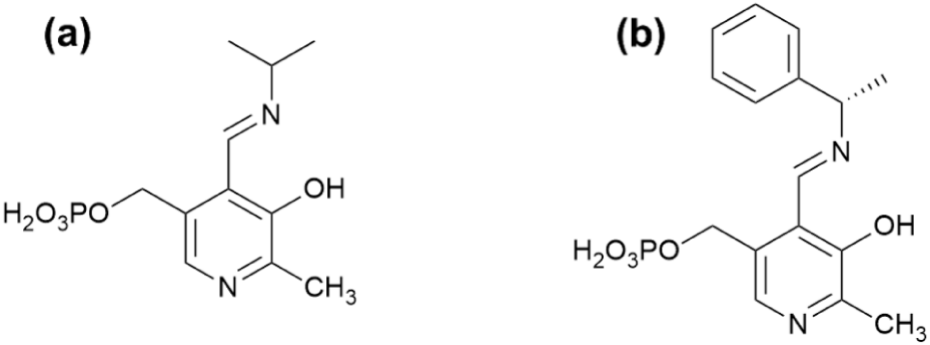
Chemical Structures of (a) Pyridoxal 5′-Phosphate Isopropylamine (PLP-IPAm) and (b) Pyridoxal 5′-Phosphate (*S*)-1-Phenylethylamine (PLP-PEA)

The study proceeds as described: following synthesis, the crystallinity of the external aldimines was assessed to determine the suitability for single-crystal X-ray diffraction, which would have enabled periodic DFT modeling. As crystals of sufficient quality could not be obtained, discrete single-molecule DFT calculations were instead performed, with the results validated using experimental inelastic neutron scattering (INS) spectroscopy. Visualization by animation software of the calculated vibrational modes was then used to support vibrational assignments for infrared and Raman spectra of PLP-IPAm and PLP-PEA. Identifying diagnostic vibrational bands associated with discrete PLP-derived bonding interactions defines a platform from which vibrational spectroscopy (e.g., Raman spectroscopy) could be applied to investigate how the representative external aldimines interact with reagents relevant to the biocatalytic synthesis of bespoke amino acids.

## 2 Experimental Section

### 2.1 Synthesis of PLP-IPAm

A 100 mL round-bottom flask was charged with a stirring bar, activated 4 Å molecular sieves and pyridoxal 5′-phosphate monohydrate (2.000 g, 7.54 mmol, 1 eq., 99%, Acros Organics). The flask was degassed with N_2_ and sealed with a Suba-Seal septum and anhydrous methanol (30 mL) was added. To the resulting suspension, isopropylamine (0.618 mL, 7.54 mmol, 1 eq., 99+%, Alfa Aesar), was added, and the resulting orange-yellow solution stirred for 24 h at room temperature. The reaction mixture was filtered over Celite, washed with methanol and dried *in vacuo* to afford the product as a yellow solid (2.073 g, yield = 95%, mp ≈ ca. 150 °C). NMR spectra (^1^H, ^13^C, ^31^P) for PLP-IPAm are provided in the Supporting Information, Figures S1 –S3.

### 2.2 Synthesis of PLP-PEA

A 100 mL round-bottom flask was charged with a stirring bar, activated 4 Å molecular sieves and pyridoxal 5′-phosphate monohydrate, (1.920 g, 7.24 mmol, 1 eq., 99%, Acros Organics). The flask was degassed with N_2_ and sealed with a Suba-Seal septum and anhydrous methanol (30 mL) was added. To the resulting suspension, (*S*)-1-phenylethylamine (0.924 mL, 7.24 mmol, 1 eq 99+%, Alfa Aesar), was added, and the resulting orange-yellow solution stirred for 24 h at room temperature. The reaction mixture was filtered over Celite, washed with methanol and dried *in vacuo* to afford the product as a yellow solid (2.216 g, yield = 87%, mp ≈ ca. 150 °C). NMR spectra (^1^H, ^13^C, ^31^P) for PLP-PEA are provided in the Supporting Information, Figures S4–S6.

### 2.3 NMR Spectroscopy

Nuclear magnetic resonance (NMR) spectra of PLP-IPAm and PLP-PEA were recorded using samples in deuterated dimethyl sulfoxide (DMSO-*d*_6_) and using a Bruker Avance III HD (400 MHz [^1^H], 101 MHz [^13^C] and 162 MHz [^31^P]). Chemical shifts (δ) are reported in parts per million (ppm). ^31^P and ^13^C NMR spectra were obtained with ^1^H decoupling. All NMR spectra were processed using Bruker Topspin 3.6.5.

### 2.4 Vibrational Spectroscopy

The INS spectra of PLP-IPAm and PLP-PEA was recorded at 20 K on the TOSCA indirect geometry spectrometer,^44^ located at the ISIS Neutron and Muon Facility of the STFC Rutherford Appleton Laboratory. INS spectra are examined in the 400–2000 cm^–1^ range, as resolution is compromised above 2000 cm^–1^ with this spectrometer. In the Supporting Information, Figures S7 and S8 present the INS spectra respectively of PLP-IPAm and PLP-PEA over an extended energy range (50–4000 cm^–1^).

ATR-IR spectra were recorded under ambient conditions in the spectroscopic range 4000–400 cm^–1^, by averaging 150 scans at a resolution of 4 cm^–1^ using a Bruker Tensor II FTIR spectrometer fitted with a Bruker A225/Q Platinum ATR unit with single reflection diamond crystal (2 mm × 2 mm); after collection of IR data, a compensating variable path length correction was applied. A deuterated triglycine sulfate (DTGS) detector and an optic set with an aperture size of 6 mm and a scanning velocity of 7.5 kHz was used for spectral acquisition.

FT-Raman spectroscopy was recorded under ambient conditions by averaging 64 scans, at a resolution of 4 cm^–1^, across the spectral range 400–4000 cm^–1^ using a Bruker MultiRam Fourier transform Raman (FT-Raman) spectrometer fitted with a germanium detector and a Nd/YAG laser using an excitation wavelength of 1064 nm. To maximize the signal-to-noise ratio, the laser power was typically 400 mW. One of the advantages of FT-Raman with near-infrared excitation is that the laser spot is large (up to 1 mm^2^), so the power density is relatively low. There is also little absorption of the laser, so heating effects are also minimal. No change in the spectra was observed with different measurement times, showing that laser damage and heating were negligible.

### 2.5 Ab Initio DFT Calculations and Vibrational Assignments

DFT calculations were performed on single-molecule models of PLP-IPAm and PLP-PEA using the Gaussian 09W software package.^45^ The structural geometry optimization and harmonic vibrational frequency calculations were carried out employing the Lee, Yang, and Parr correlation functional, in conjunction with the Becke’s local three-parameter hybrid exchange functional (B3LYP),^46,47^ and a 6-311G++(d,p) basis set.^48^ Computed vibrational frequencies and intensities calculated were used to generate simulated INS spectra using AbINS,^49^ within the Mantid software suite.^50^ To assist in the assignment of the ATR-IR and FT-Raman spectra of PLP-IPAm and PLP-PEA, the predicted vibrational data set was used to simulate the IR and Raman spectra, and atomic displacements for both compounds using GaussView 5.0.9 ^45^ (Figure S9).

Visualization of the predicted vibrational modes revealed that multiple atomic displacements often contribute to a single predicted wavenumber, reflecting significant coupling between vibrational motions. Consequently, assignments were made using an “approximate descriptive” approach, whereby each calculated normal mode was assigned with the most pronounced atomic displacement observed.^51–53^ The assignment of monosubstituted benzene moieties is based on a nomenclature introduced by Gardner and Wright,^54^ which presents a simplified notation scheme to describe the molecular vibrations of a monosubstituted benzene ring (**ESI**, Figure S10 and Table S1). Furthermore, seminal sources such as those by Lin-Vien et al.,^55^ have also been consulted to support additional group frequency assignments.

Visualization of low-energy modes detected below 400 cm^–1^ reveals a complex mixture of skeletal deformation modes and lattice modes, which have not been considered for assignment in this communication. It should be noted that the predicted vibrational wavenumbers have not been adjusted using a scaling factor correction,^56^ to enable direct comparison with the experimental spectra. Comprehensive vibrational assignments of PLP-IPAm and PLP-PEA from 400 to 4000 cm^–1^ are presented in the Supporting Information, Tables S2 and S3.

## 3 Results and Discussion

### 3.1 Synthesis of PLP-IPAm and PLP-PEA

A variety of synthetic routes for PLP-derived imines are documented.^57–60^ PLP-IPAm and PLP-PEA were synthesized using a modified condensation procedure reported by Pilicer et al.^60^ Initial attempts to synthesize both compounds using 5 equiv of either IPAm, or PEA, revealed significant amounts of residual amine in the obtained product. The synthetic route was refined by reducing the quantity of IPAm and PEA to 1 equiv and introducing molecular sieves to dehydrate the resulting reaction mixture. Trace amounts of amine were efficiently removed by thorough high-vacuum drying, resulting in products of high purity. The modified procedure was successfully scaled up to synthesize 2–5 g of both PLP-IPAm and PLP-PEA. This increase in production quantity was necessary to obtain adequate amounts of each material for INS experiments (0.5–2.0 g for a hydrogenous sample).^61^

### 3.2 Ab Initio DFT Validation via INS

Initial powder X-ray diffraction (PXRD) analysis of PLP-IPAm and PLP-PEA confirmed that both samples were amorphous, with no evidence of microcrystallinity (Figure S11). This lack of long-range order precluded the use of single-crystal or powder-based X-ray and neutron diffraction methods for structure determination. Multiple recrystallization strategies were explored to obtain single crystals suitable for diffraction analysis; however, these efforts were unsuccessful. A summary of the conditions screened is provided in the Supporting Information, along with a representative PXRD pattern from a postrecrystallization attempt, which further confirms the absence of crystalline material (Figure S12). Due to the lack of single-crystal structures, periodic-DFT calculations, ideal for accurately predicting vibrational properties in the solid state by accounting for intermolecular interactions and crystal packing, were unfeasible. Consequently, discrete DFT calculations were employed to assist in assigning the ATR-IR and FT-Raman spectra collected for both PLP-IPAm and PLP-PEA.

An assessment of the suitability of the resulting DFT outputs was undertaken by comparing simulated INS spectra, derived from the predicted vibrational data sets, with experimentally obtained INS spectra (Figures 1 and 2). Validation of the computational data sets via INS spectroscopy is a well-established methodology.^62–66^ The DFT-optimized structures of PLP-IPAm and PLP-PEA are shown in Figure 3. Qualitative comparisons of the simulated and experimental INS spectra for each compound show strong agreement in peak positions and relative intensities, enabling confident vibrational assignments of the optical vibrational spectra while underscoring the technique’s sensitivity to hydrogen-rich moieties. Within PLP-IPAm and PLP-PEA, many of these functionalities correspond to the compound-specific isopropyl and 1-phenylethyl, respectively. Analysis of the INS data provides useful assignments that complement optical vibrational spectroscopy. Methyl-associated vibrational features appear in the INS spectra of both PLP-IPAm and PLP-PEA, reflecting their shared PLP scaffold. However, these bands are markedly more intense in the PLP-IPAm spectrum, consistent with its higher methyl content. Specifically, the band at 948 cm^–1^ involves rocking motions, the band at 1378 cm^–1^ is due to symmetric deformations, and the band at 1456 cm^–1^ corresponds to asymmetric deformations of the methyl substituents. In contrast, the INS spectrum of PLP-PEA exhibits a more complex vibrational profile arising from multiple modes associated with the phenyl ring functionality. Several prominent features in the low- to midfrequency range can be confidently assigned based on simulated normal modes. A band at 404 cm^–1^ corresponds to an M _14_(a_2_) out-of-plane ring deformation, while the band at 619 cm^–1^ is assigned to an M_29_(b_2_) in-plane stretching vibration. Multiple signals related to out-of-plane deformations ( M _18_(b_1_), M_17_(b_1_), M _13_(a_2_) and M _16_(b_1_)) are observed between 900 and 700 cm^–1^. A feature at 1075 cm^–1^ is assigned to a M _28_(b_2_) in-plane ring deformation, while the band at 1182 cm^–1^ arises from M _27_(b_2_) and M _7_(a_1_), both corresponding to in-plane deformation modes.

**Figure 1:**
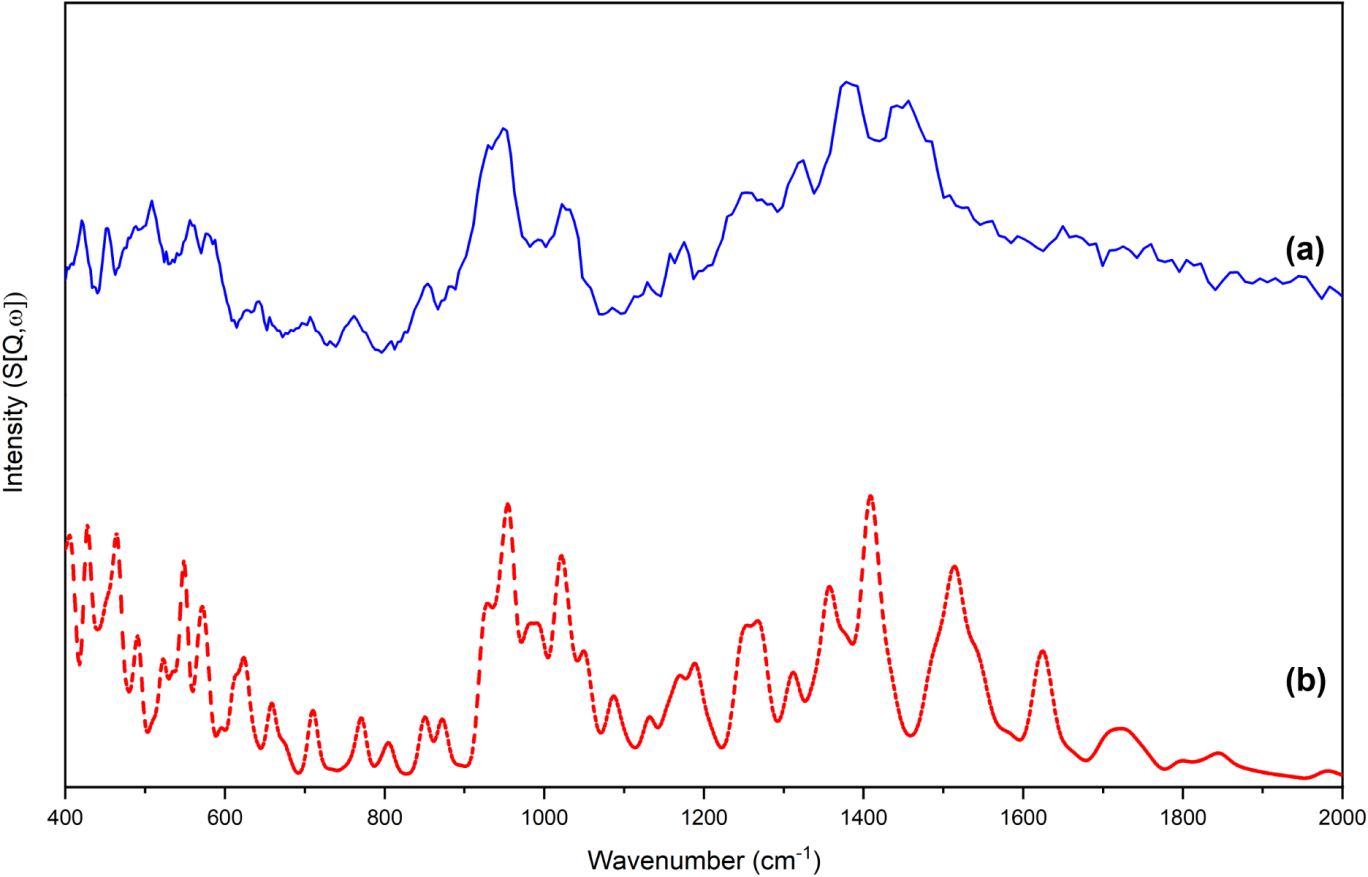
(a) Experimental and (b) simulated INS spectra in the range 400–2000 cm^–1^ of PLP-IPAm.

**Figure 2:**
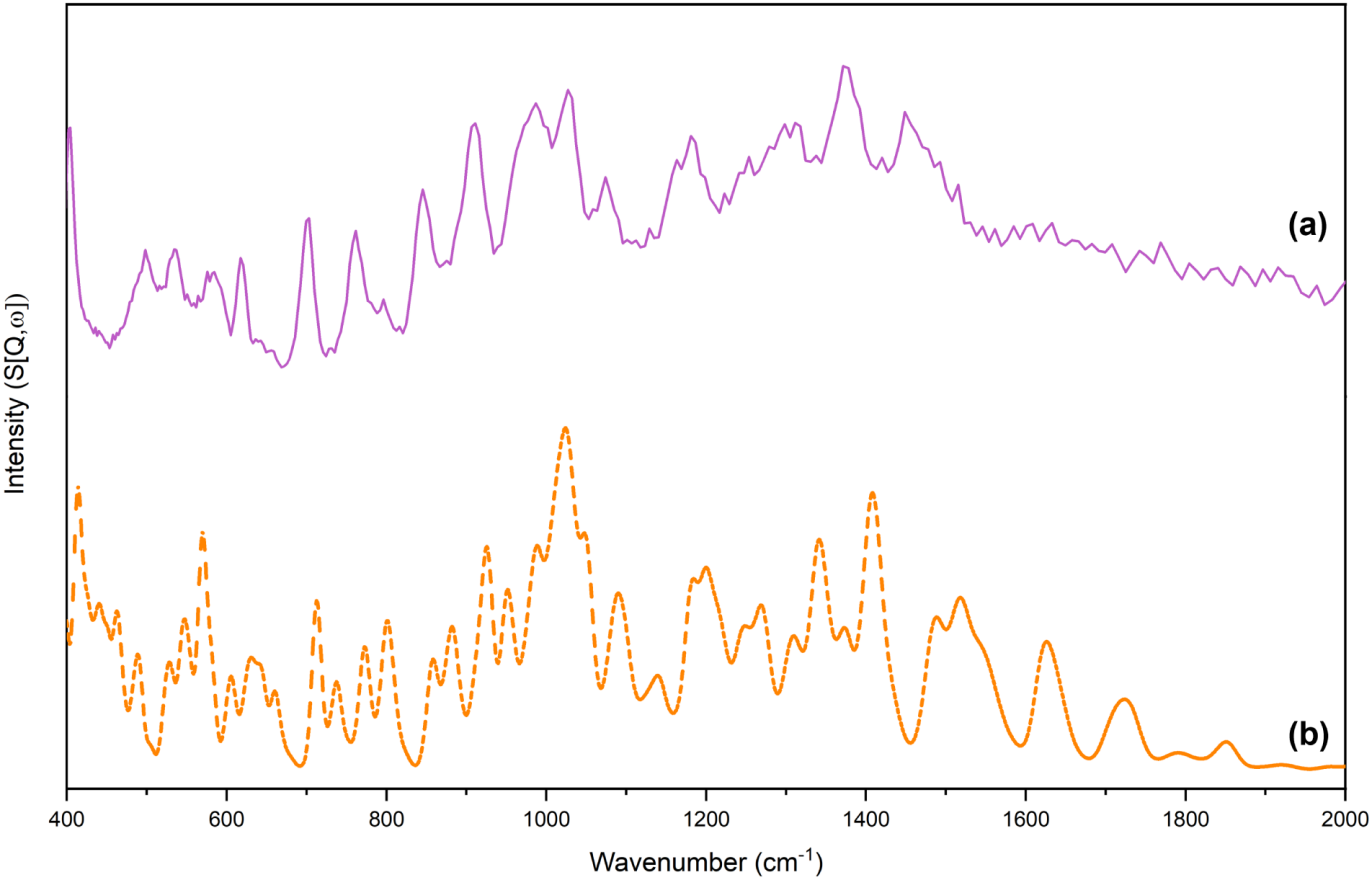
(a) Experimental and (b) simulated INS spectra in the range 400–2000 cm^–1^ of PLP-PEA.

**Figure 3:**
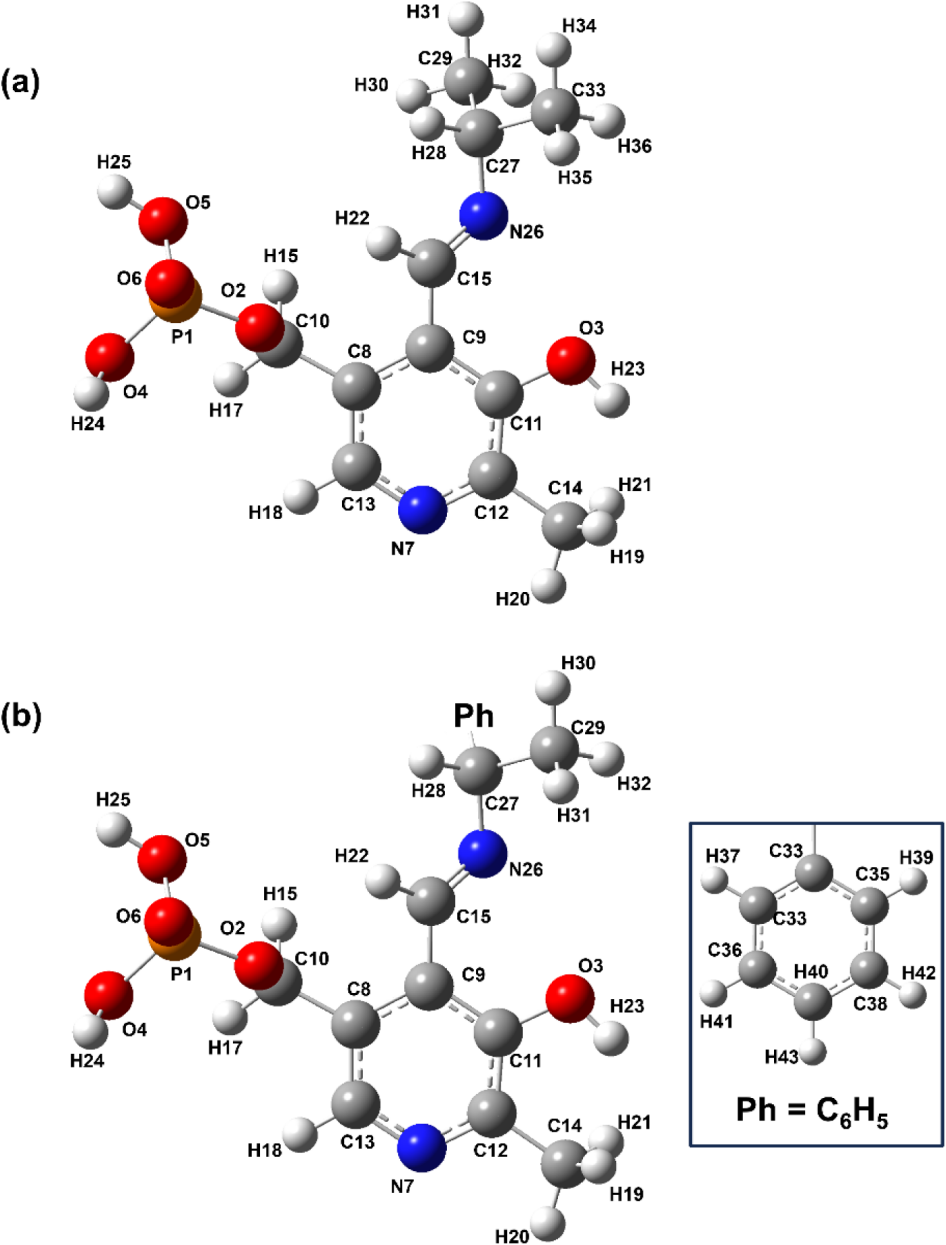
Chemical structures of (a) pyridoxal 5′-phosphate isopropylamine and (b) pyridoxal 5′-phosphate (S)-1-phenylethylamine obtained from Gaussian optimization calculations using the B3LYP/6-311G++(d,p) basis set. Atom numbering corresponds to their positions in the molecule.

INS spectra are routinely measured below 20 K to minimize the effect of the Debye–Waller factor.^59^ A question arises as to whether this will modify the hydrogen-bonding present. The usual result of lowering the temperature is to decrease the lattice parameters, thus squeezing the molecules closer together. This is likely to increase the strength of the hydrogen-bonding, resulting in a downshift of the O–H stretch modes and an upshift of the deformation modes. Such a change in the structure will manifest in all three forms of spectroscopy. We note that for the strongly hydrogen-bonded system LiOH·H_2_O,^67^ the O–H stretch is at the same energy in the room temperature infrared and Raman spectra as it is in the 20 K INS spectrum.

### 3.3 Analysis of the ATR-IR Spectra of PLP-IPAm and PLP-PEA

The ATR-IR spectra of PLP-IPAm and PLP-PEA, shown in Figure 4, present a significant degree of commonality in the fingerprint region, reflecting the shared PLP-based structural framework of both compounds. A notable distinction within this region involves the identification of the *ν*(C=N) stretching mode associated with the imine linkage present in both compounds. This mode, predicted at 1713 cm^–1^ for PLP-IPAm, is absent in its ATR-IR spectrum (Figure 4a), whereas it appears at 1688 cm^–1^ in the ATR-IR spectrum of PLP-PEA as a medium intensity band (Figure 4b). In the higher energy stretching region, the ATR-IR spectrum of PLP-IPAm features low intensity bands at 2873 and 2925 cm^–1^, corresponding to symmetric and asymmetric stretching from the pyridine methyl group, as well as symmetrical stretching contributions from the bridging methylene group, which links the pyridine ring to the phosphate group.

**Figure 4:**
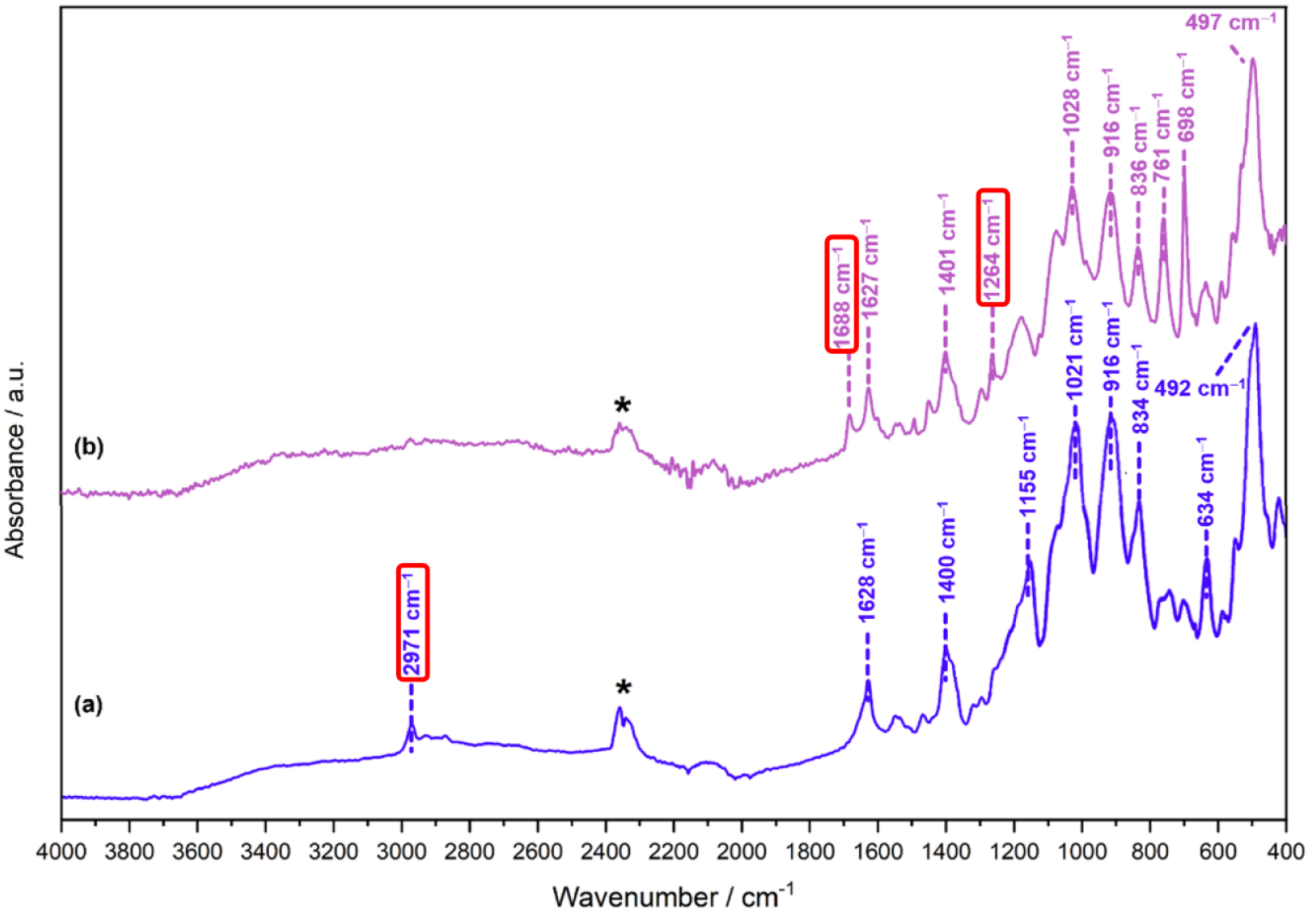
ATR-IR spectrum of (a) PLP-IPAm and (b) PLP-PEA in the range 4000–400 cm^–1^. The red boxed wavenumber values highlight diagnostic modes mentioned in the text. The asterisk at ca. 2300 cm^–1^ represents ambient CO_2_.

A medium intensity band at 2971 cm^–1^, attributed to asymmetric methyl stretching of the isopropyl group serves as a structural marker for PLP-IPAm. In contrast, the PLP-PEA spectrum is largely featureless in this region, with no identifiable peaks observed. Although DFT calculations for PLP-PEA predict bands associated with symmetric and asymmetric methyl stretching in the 1-phenylethyl group at 3002 and 3047 cm^–1^ respectively, and a symmetrical stretching mode for the methylene group at 3061 cm^–1^, none of these calculated modes are experimentally pronounced. The low-intensity bands in the PLP-IPAm spectrum and the lack of observable peaks in the PLP-PEA spectrum are indicative of possible influencing hydrogen-bonding interactions and anharmonic effects within the amorphous sample matrix.^67,68^ The absence of the 2971 cm^–1^ band in PLP-PEA further reinforces its assignment as a signature feature for the isopropyl group, distinguishing PLP-IPAm from PLP-PEA.

As discussed in Section 3.2, comprehensive vibrational assignments for the ATR-IR spectra of PLP-IPAm and PLP-PEA are provided in the Supporting Information (Tables S1 and S2). Given the multifunctional nature of both molecules, these data sets are necessarily extensive. To support interpretation of the IR spectra, Table 1 presents a cursory summary of selected principal vibrational modes for each compound. The following discussion draws upon both Table 1 and the full assignments in Tables S1 and S2.

**Table 1:** Notable Vibrational Modes Present within the ATR-IR Spectra of PLP-IPAm and PLP-PEA; Pyr = Pentasubstituted Pyridine; ***ν***_**s**_ = Symmetric Stretch, ***ν***_**as**_ = Asymmetric Stretch, ***δ*** = Deformation, ***δ***_s_ = Symmetric Deformation, *ω* = Wag, ipb = In-Plane Bend, opb = Out-Of-Plane; *m* = Medium, *s* = Strong, *vs* = Very Strong

	ATR-IR/cm^–1^	
Approximate description	PLP-IPAm	PLP-PEA
*δ*_s_(O–P–O),*ρ*(Pyr)	492 vs	497 *s*
*δ*(Pyr)opb (both); M _29_(b_2_) (PLP-PEA)	634 *s*	632 *m*
*δ*(Pyr)ipb (both); M _18_(b_1_) (PLP-PEA)	701 *m*	698 *s*
*δ*(Pyr)opb (both); M _17_(b_1_) (PLP-PEA)	764 *m*	761 *s*
*ν*_s/as_(O–P–O) (both); M _13_(a_2_) (PLP-PEA)	834 *s*	836 *s*
*δ*_Pyr_(C–H)opb (both);*ρ*(CH_3_) (PLP-IPAm); M _16_(b_1_) (PLP-PEA)	916 vs	916 vs
*δ*(P–OH)opb/ipd (both); M _9_(a_1_), M _15_(b_1_) (PLP-PEA)	1021 vs	1028 vs
*ν*(Pyr),*δ*(Pyr)ipb	1155 *s*	-
*ν*(Pyr),*δ*(Pyr)ipb	-	1264 *s*
*δ*_s_(CH_3_),*ω*(CH_2_),*δ*(Pyr)ipb	1400 *s*	1401 *s*
*ν*(Pyr) (both), M _4_(a_1_), M _23_(b_2_) (PLP-PEA)	1628 *s*	1627 *s*
*ν*(C=N)	-	1688 *s*
ν_as_(CH_3_),ν_as_(CH_2_)	2971 *m*	-

#### 3.3.1 Phosphate Group Vibrations

The experimental ATR-IR spectra of PLP-IPAm and PLP-PEA display vibrational features characteristic of the phosphate moiety common to PLP-derived external aldimines. PLP-IPAm and PLP-PEA exhibit absorptions between 492–422 cm^–1^ and 497 cm^–1^, respectively, corresponding to in-phase and out-of-phase *δ*(O–P–O) bending modes. These features also include a pyridine ring rocking vibration (*ρ*(Pyr)) in both compounds, arising from a concerted displacement of ring atoms, which complicates precise attribution to individual atomic motions. A shared vibrational signature in both spectra corresponds to the symmetric and asymmetric O–P–O stretching modes at 834 cm^–1^ and 836 cm^–1^, respectively. Strong signals at 1021 cm^–1^ for PLP-IPAm and 1028 cm^–1^ for PLP-PEA are assigned to the in-phase and out-of-phase *δ*(O–H) bending modes of the phosphate group.

#### 3.3.2 Pyridine Ring and C–H Vibrations

Both ATR-IR spectra exhibit several moderately to highly intense bands associated with out-of-plane pyridine ring deformations. In PLP-IPAm, these are observed at 632 cm^–1^ and 761 cm^–1^, while in PLP-PEA, analogous modes occur at 634 cm^–1^ and 764 cm^–1^. Additional in-plane pyridine ring deformation modes (*δ*(Pyr)­ipb) are present at 701 cm^–1^ in PLP-IPAm and 698 cm^–1^ in PLP-PEA, although the precise vibrational contributions to these features are difficult to discern due to overlapping motions. Distinct pyridine ring stretching and in-plane deformation modes are also identified in each compound: for PLP-IPAm, a characteristic signal appears at 1155 cm^–1^, while for PLP-PEA, a corresponding feature is observed at 1264 cm^–1^. These sets of bands are attributed to different atomic displacement patterns within the pyridine ring of each molecule. Additionally, an in-plane ring stretching vibration is evident at 1627 cm^–1^ in PLP-IPAm and at 1628 cm^–1^ in PLP-PEA.

Beyond pyridine-associated features, several vibrational bands in the ATR-IR spectra of both compounds are linked to C–H bending and rocking motions. Notably, intense absorptions at 916 cm^–1^ are assigned to methyl rocking vibrations originating from substituents on both the pyridine ring and the R-groups of PLP-IPAm and PLP-PEA. Broad, intense bands at 1400 cm^–1^ (PLP-IPAm) and 1401 cm^–1^ (PLP-PEA) are influenced by symmetric *δ*(CH_3_) vibrations of the methyl groups, as well as a wagging mode of the bridging −CH_2_– group and additional in-plane pyridine deformations, as supported by visualization of the simulated spectra. Furthermore, in PLP-IPAm, the band at 1466 cm^–1^ is attributed to methyl twisting and asymmetric bending vibrations. A similar feature is observed at 1493 cm^–1^ in PLP-PEA, reflecting analogous dynamic behavior in both molecules.

#### 3.3.3 Monosubstituted Benzene Ring Modes

The ATR-IR spectrum of PLP-PEA exhibits several characteristic deformation and stretching features associated with the monosubstituted benzene functionality. A signal at 556 cm^–1^ corresponds to the M _19_(b_1_) out-of-plane ring deformation, while the feature at 632 cm^–1^ is attributed to the M _29_(b_2_) in-plane ring stretching mode. Relatively intense absorptions at 698 cm^–1^ and 761 cm^–1^ are assigned to the M _18_(b_1_) and M _17_(b_1_) out-of-plane deformation modes, respectively. A further vibration at 836 cm^–1^ is linked to the M _13_(a_2_) out-of-plane deformation. The M _16_(b_1_) out-of-plane deformation mode contributes to the strong signal observed at 916 cm^–1^. Both the M _9_(a_1_) in-plane and M _15_(b_1_) out-of-plane deformation modes contribute to the prominent absorption at 1028 cm^–1^. The M _26_(b_2_) in-plane ring stretch is associated with the 1297 cm^–1^ feature, while the M _5_(a_1_) and M _24_(b_2_) in-plane stretching modes are responsible for the signals at 1493 cm^–1^ and 1549 cm^–1^, respectively. Finally, a strong absorption at 1627 cm^–1^ arises from contributions of the M _4_(a_1_) and M _23_(b_2_) symmetric in-plane ring stretching vibrations.

### 3.4 Analysis of the FT-Raman Spectra of PLP-IPAm and PLP-PEA

The FT-Raman spectra of PLP-IPAm and PLP-PEA, presented in Figure 5, exhibit several shared spectral features, consistent with the common PLP-based structural framework of both compounds. As in the ATR-IR spectra (Figure 4), the lower-energy fingerprint region reveals a high degree of similarity between the two spectra. Conversely, unlike the ATR-IR spectra, where the high-energy stretching region is either featureless or exhibits poorly resolved low-intensity peaks, the corresponding region in the FT-Raman spectra contains several well-resolved bands that can be confidently assigned. Accordingly, the following discussion examines both the vibrational features common to the two spectra and those specific to each compound. Principal spectral distinctions arise from the structural divergence between the IPAm and PEA functional groups in PLP-IPAm and PLP-PEA, respectively, with the degree and nature of spectral convergence or divergence depending on the specific vibrational region of the FT-Raman spectrum under consideration.

**Figure 5:**
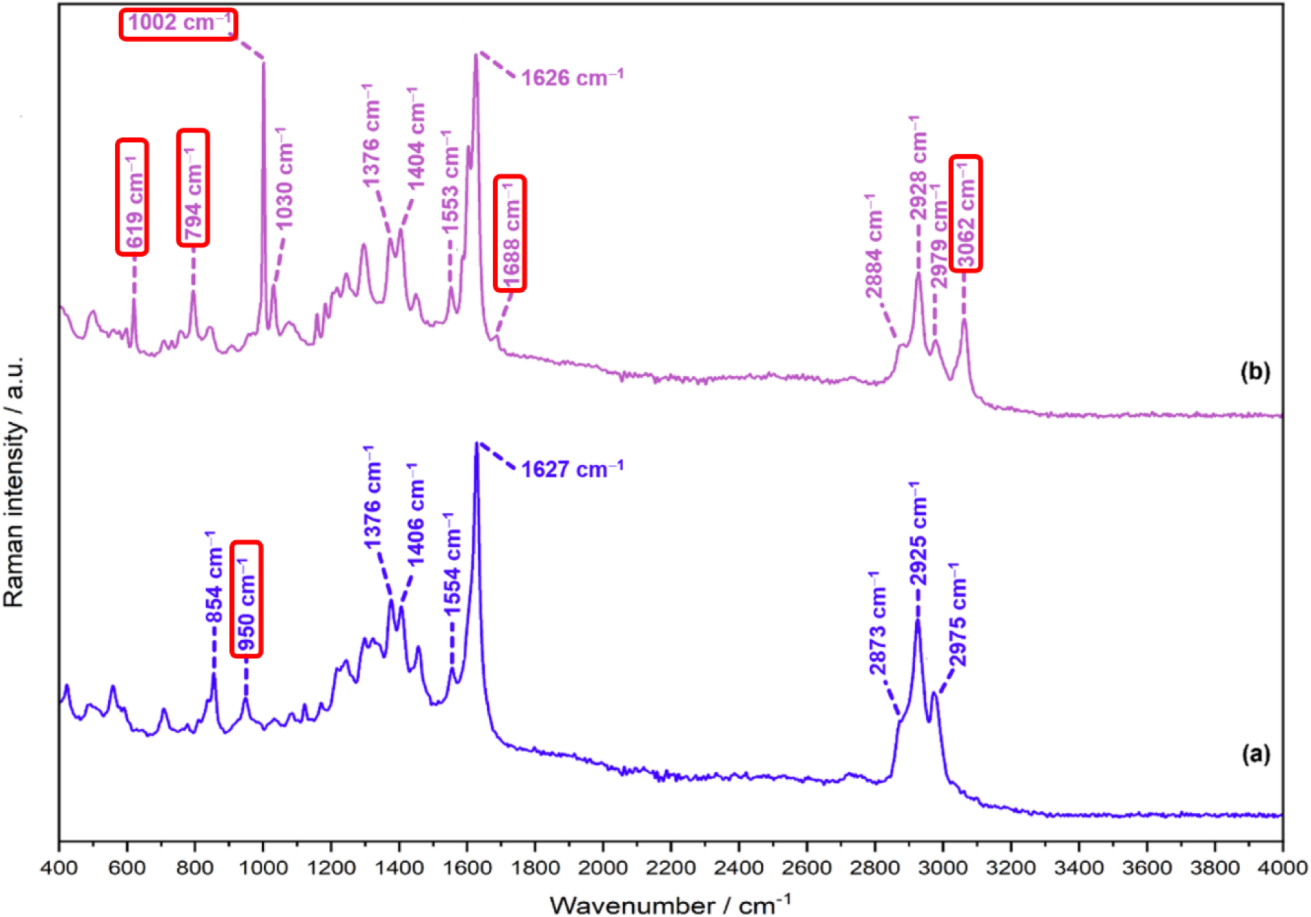
FT-Raman spectrum of (a) PLP-IPAm and (b) PLP-PEA in the range 400–4000 cm^–1^. The red boxed wavenumber values highlight diagnostic modes mentioned in the text.

Supplementing the comprehensive vibrational assignments for the ATR-IR and FT-Raman spectra of PLP-IPAm and PLP-PEA provided in the ESI (Tables S2 and S3), Table 2 presents a concise summary of selected key Raman active vibrational modes for each compound.

**Table 2:** Notable Vibrational Modes Present within the FT-Raman Spectra of PLP-IPAm and PLP-PEA. Pyr = Pentasubstituted Pyridine; ***ν***_s_ = Symmetric Stretch, ***ν***_as_ = Asymmetric Stretch, ***δ*** = Deformation, ***δ***_s_ = Symmetric Deformation, ***ω*** = Wag, ipb = In-Plane Bend, opb = Out-Of-Plane; *m* = Medium, *s* = Strong, *vs* = Very Strong

	FT-Raman/cm^–1^	
Approximate description	PLP-IPAm	PLP-PEA
*δ*(Pyr)opb, M _29_(b_2_)	–	619 *m*
*δ*(Pyr)opb, M _10_(a_1_)	–	794 *s*
*ν*_s/as_(O–P–O), M _13_(a_2_) (PLP-PEA)	854 *m*	845 *m*
*ρ*(CH_3_)	950 *m*	–
*ρ*(CH_3_), M _9_(a_1_), M _12_(a_2_), M _15_(b_1_)	–	1002 vs
*δ*(P–OH)opb/ipd (both); M _8_(a_1_) (PLP-PEA)	1030 *w*	1030 *m*
*δ*_s_(CH_3_) (PLP-IPAm); M _25_(b_2_), M _26_(b_2_) (PLP-PEA)	1376 *s*	1375 *s*
*ω*(CH_2_),*δ*_s_(CH_3_),*δ*(C–H)	1406 *s*	1404 *s*
*ν*(Pyr) (both); M _5_(a_1_) (PLP-PEA)	1554 *m*	1553 *m*
*ν*(Pyr) (both), M _4_(a_1_), M _23_(b_2_) (PLP-PEA)	1627 vs	1626 vs
*ν*(C=N)	–	1688 *m*
*ν*_Pyr_(C–H)	2873 *sh*	2884 *sh*
*ν*(C–H),*ν*_s/as_(CH_3_),*ν*_s_(CH_2_)	2925 vs	2928 *s*
*ν*(C–H),*ν*_as_(CH_3_),*ν*_as_(CH_2_)	2975 *s*	2979 *m*
M_1_(a_1_), M _2_(a_1_), M _3_(a_1_), M _21_(b_2_), M _22_(b_2_)	–	3062 *s*

#### 3.4.1 Unique Low-Frequency (2000–400 cm^–1^) FT-Raman Modes of PLP-IPAm and PLP-PEA

The FT-Raman spectrum of PLP-IPAm exhibits several peaks of reasonable intensity that warrant detailed analysis. A weak but distinct band at 590 cm^–1^ is associated with two vibrational components: an out-of-plane deformation of the pyridine ring, which can be readily assigned to specific atoms, and an in-plane pyridine deformation (*δ*(Pyr)), for which visual inspection does not allow unambiguous identification of the atoms involved. A medium-intensity band at 950 cm^–1^ corresponds to a distinct vibrational mode attributed to isopropyl methyl rocking modes. These vibrational assignments are consistent with the molecular structure of PLP-IPAm.

Comparatively, the FT-Raman spectrum of PLP-PEA reveal notably distinctive peaks characterized by several intense bands primarily associated with monosubstituted benzene and pyridine ring vibrations. A prominent peak at 619 cm^–1^ is assigned to the M_29_(b_2_) out-of-plane ring deformation mode, alongside in-plane pyridine ring deformations. Another significant band at 794 cm^–1^ corresponds to the M_10_(a_1_) in-plane ring deformation mode and an out-of-plane pyridine ring deformation. Additionally, the PLP-PEA spectrum exhibits an intense feature at 1002 cm^–1^, attributed to isopropyl methyl rocking modes, M_9_(a_1_) in-plane ring breathing, and M_12_(a_2_) and M_15_(b_1_) out-of-plane deformations. A diagnostic peak at 1688 cm^–1^, corresponding to the *ν*(C=N) mode, is again exclusively observed in the FT-Raman spectrum of PLP-PEA. Although the intensity of this peak varies between ATR-IR and FT-Raman spectra of PLP-PEA, its consistent identification provides a valuable spectroscopic marker for distinguishing PLP-PEA from PLP-IPAm.

The absence of the *ν*(C=N) stretching mode in the ATR-IR and FT-Raman spectra of PLP-IPAm prompted further investigation through computational modeling. To evaluate the potential influence of intramolecular hydrogen bonding, two alternative isolated-molecule DFT models were generated: one incorporating a neutral hydrogen bond and the other a zwitterionic hydrogen-bonding motif; both options feature an internal hydrogen bond stabilizing a six-membered ring (Supporting Information), Figure S13. In both cases, the calculated ν­(C=N) stretching frequencies were red-shifted (from 1713 cm^–1^) to 1686 cm^–1^ and 1680 cm^–1^ respectively and exhibited reduced spectral definition. Although it is not possible to discern whether the charged (Figure S13a) or uncharged (Figure S13b) structures are more representative of the experimental case, the findings suggest that intramolecular hydrogen bonding in PLP-IPAm perturbs the local electronic environment of the imine group, leading to band broadening or attenuation that renders the *ν*(C=N) vibration unresolved in the experimental spectra. This outcome highlights a subtle yet significant structural divergence between PLP-IPAm and PLP-PEA and underscores the value of complementary theoretical models in interpreting spectroscopic data of structurally flexible, noncrystalline systems.

#### 3.4.2 Shared Low-Frequency (2000–400 cm^–1^) FT-Raman Modes

The FT-Raman spectra of PLP-IPAm and PLP-PEA exhibit broadly comparable vibrational profiles, particularly within the fingerprint region, where multiple bands occur at closely aligned positions. This spectral similarity reflects analogous molecular environments. Although certain bands incorporate additional vibrational contributions, the close wavenumber alignment justifies discussion within a shared vibrational framework. Both spectra feature bands at 854 cm^–1^ (PLP-IPAm) and 845 cm^–1^ (PLP-PEA), attributed to asymmetric and symmetric *ν*(O–P–O) stretching modes. In the PLP-PEA spectrum, this feature also includes a contribution from the M_13_(a_2_) out-of-plane deformation mode. A band at 1030 cm^–1^ is observed in both spectra and is associated with in-plane and out-of-plane *δ*(OH) deformations from the phenolic moieties of the phosphate group. For PLP-PEA, this mode is further supplemented by the M_8_(a_1_) in-plane deformation. Bands at 1299 cm^–1^ (PLP-IPAm) and 1296 cm^–1^ (PLP-PEA) are assigned to *ν*(P=O) stretching modes, coupled with in-plane pyridine ring C–H bending.

A band observed at 1375 cm^–1^ in PLP-IPAm and 1376 cm^–1^ in PLP-PEA is present in both spectra. For PLP-IPAm, this mode is assigned to out-of-phase symmetrical isopropyl methyl deformation vibrations, while for PLP-PEA, it is attributed to the M_25_(b_2_) and M_26_(b_2_) in-plane ring stretching vibrations. In-phase symmetric isopropyl methyl deformation modes, CH_2_ wagging modes, and out-of-phase C–H deformation modes from the imine carbon and the methine carbon appear at 1406 cm^–1^ and 1404 cm^–1^, respectively. Additionally, both compounds display intense bands around 1554 cm^–1^, consistent with in-plane pyridine ring stretching. In PLP-PEA, this band also receives contributions from the M_5_(a_1_) in-plane ring stretching mode. Finally, peaks at 1627 cm^–1^ (PLP-IPAm) and 1626 cm^–1^ (PLP-PEA) are assigned to in-plane pyridine ring stretching. In the PLP-PEA spectrum, this feature additionally includes contributions from the M_4_(a_1_) and M_23_(b_2_) modes, both associated with in-plane ring stretching.

#### 3.4.3 Aliphatic Stretching Modes in the High-Frequency (4000–2000 cm^–1^) FT-Raman Region

The higher energy stretching region of the FT-Raman spectra reveals several shared spectral features, reflecting the overall similarity between the band profiles of PLP-IPAm and PLP-PEA. Both spectra exhibit a shared cluster of three bands between 2980–2870 cm^–1^, corresponding to aliphatic C–H stretching vibrations. The peak at 2873 cm^–1^ for PLP-IPAm and at 2884 cm^–1^ in PLP-PEA is attributed to methine C–H stretching. Additionally, peaks of similar wavenumber are observed at 2925 cm^–1^ for PLP-IPAm and 2928 cm^–1^ for PLP-PEA. Visualization of the calculated vibrational modes corresponding to these bands reveals contributions from a number of modes: (i) symmetric stretching modes of the methyl groups associated with the R groups in both external aldimines; (ii) symmetric, and asymmetric stretching modes associated with the pyridine ring methyl group; (iii) C–H stretching vibrations associated with the imine carbon in both PLP-IPAm and PLP-PEA; and (iv) symmetric methylene stretching. Interestingly, the intensity of PLP-IPAm’s peak at 2925 cm^–1^ is significantly greater than that of the corresponding peak at 2928 cm^–1^ in PLP-PEA. As suggested for the spectral intensity differences observed in the INS spectra (Section 3.2), the enhanced intensity may arise from additional contributions associated with symmetric methyl stretching vibrations of the isopropyl group.

The highest intensity feature in both spectra appears at 2975 cm^–1^ for PLP-IPAm and at 2979 cm^–1^ for PLP-PEA. In both compounds, these bands predominantly involve asymmetric stretching of the methyl groups within the isopropyl and 1-phenylethyl moieties of their respective external aldimines, as well as asymmetric stretching of the methyl group on the pyridine ring. Additional contributions arise from asymmetric stretching of the methylene bridge connecting the pyridine ring and phosphate group, and a *ν*(C–H) stretching vibration of the pyridine ring. It is also notable that despite the presence of hydroxyl groups on both the pyridine ring and in phosphate moiety, no O–H stretching bands are observed experimentally in the FT-Raman spectra of either PLP-PEA or PLP-IPAm. While DFT simulations predict intense O–H stretching features above 3800 cm^–1^, their absence in the experimental Raman spectra is not unusual. Indeed, the weakness of the O–H stretch is one of the reasons why Raman spectra are commonly measured in aqueous solution. Our DFT calculations are for the isolated molecule, so the intermolecular interactions that are at the heart of hydrogen-bonding are absent from our model, and the O–H stretch modes are calculated too high in energy. In addition, the O–H stretch is notoriously anharmonic, so this will also shift it to lower energy, an effect that is also not included in the calculations. In addition, the response of the Ge detector used here falls off rapidly above ∼3400 cm^–1^ making observation of O–H stretch modes challenging.^68^

#### 3.4.4 Diagnostic Aromatic Modes in the High-Frequency (4000–2000 cm^–1^) FT-Raman Spectrum of PLP-PEA

The higher energy region of the FT-Raman spectrum of PLP-PEA exclusively presents a prominent peak at 3062 cm^–1^, corresponds to a set of aromatic C–H stretching modes ( M _1_(a_1_), M _2_(a_1_), M _3_(a_1_), M _21_(b_2_) and M _22_(b_2_)) associated with the monosubstituted benzene ring moiety, highlighting the 1-phenylethyl group of PLP-PEA as a distinct spectroscopic handle for identifying aromatic substitution in the external aldimine moiety. The sharpness and intensity of this band confirms its assignment to aromatic stretching modes rather than any contribution from hydroxyl groups, the absence of which in this region has already been discussed (Section 3.4.3).

## 4 Conclusions

Two model external aldimines, PLP-IPAm and PLP-PEA, were synthesized and characterized using vibrational spectroscopy. Single-molecule DFT calculations were used to simulate their ATR-IR and FT-Raman spectra, and these predictions were validated through comparison with experimental INS data. Animated vibrational mode visualizations derived from the DFT outputs enabled assignment of key spectral features, facilitating detailed vibrational assignments for both compounds.

Although the ATR-IR spectra of PLP-IPAm and PLP-PEA (Figure 4) exhibit significant similarities due to their common PLP scaffold, Table 1 shows that each compound also displays distinctive vibrational features that serve as diagnostic markers for both shared functional groups and compound-specific motifs. Notable shared features include characteristic vibrational modes of the pyridine ring and phosphate moiety, such as δ­(O–P–O) bending, symmetric and asymmetric O–P–O stretching, and O–H bending. These phosphate group vibrations are particularly noteworthy, as they highlight regions of the molecule likely to participate in hydrogen bonding and electrostatic interactions within enzyme active sites, reinforcing their mechanistic relevance in PLP-dependent catalysis.

Superimposed on this common spectral profile are distinct bands that differentiate the two aldimines: PLP-PEA displays monosubstituted benzene ring modes, whereas PLP-IPAm exhibits isopropyl-specific features, including a medium-intensity band at 2971 cm^–1^ attributed to asymmetric methyl stretching, along with characteristic C–H bending and rocking vibrations. Together, these features provide spectroscopic handles that allow for differentiation between the two Schiff bases. Furthermore, it is useful to consider the prospects of whether vibrational assignments of the phenolic group of PLP could be useful for monitoring the protonation state of the group. While it is possible to protonate phenol and substituted phenols at the oxygen atom, such an action requires the use of superacids so, under physiological conditions, this scenario will not occur. However, protonation of either (or both) of the pyridine or exocyclic nitrogen atoms of PLP will result in significant changes in the spectra. These will be especially apparent in the infrared spectrum, where strong N–H stretch modes will occur around 3200–3400 cm^–1^.

The FT-Raman spectra (Figure 5), while broadly similar due to the shared molecular scaffold, also reveal compound-specific markers that serve as reliable spectroscopic discriminants (Table 2). Key differentiators include isopropyl-associated methyl rocking modes in PLP-IPAm, a prominent aromatic C–H stretching band at 3062 cm^–1^ in PLP-PEA, and a well-defined *ν*(C=N) band in PLP-PEA that is absent in PLP-IPAm; likely suppressed by intramolecular hydrogen bonding, as supported by DFT modeling. Compared to ATR-IR, FT-Raman spectroscopy proved effective in resolving high-frequency stretching regions, enabling assignments where IR spectra were featureless or poorly resolved. Interestingly, the predicted O–H stretching bands (above 3800 cm^–1^) were not observed in either compound’s Raman spectrum, likely due to hydrogen bonding interactions that red-shift and broaden these modes beyond detection. This observation underscores the importance of local noncovalent interactions in shaping vibrational behavior in amorphous systems. Overall, FT-Raman spectroscopy has demonstrated diagnostic potential in the characterization of flexible PLP-derived external aldimines, offering structural insights that extend and complement conventional IR analysis.

The comprehensive vibrational assignments compiled in Tables S2 and S3 provide a valuable foundation for probing specific molecular interactions and show that vibrational spectroscopy could be deployed to discriminate between structurally distinct PLP derivatives. For example, in the infrared, PLP-PEA uniquely exhibits a ν­(C=N) mode at 1688 cm^–1^ and PLP-IPAm displays CH_3_ and CH_2_ stretching features about 2971 cm^–1^. Although the precise nature of external aldimine binding to amino acid substrates remains to be fully elucidated, the assignments established in this study offer a robust framework for future mechanistic investigations of PLP-dependent catalysis.

Given its compatibility with aqueous environments, Raman spectroscopy is well-suited for both laboratory and industrial contexts. For the solid-state Raman spectra shown in Figure 5, with the same measurement time, 10% of the sample size should be detectable. Assuming a laser spot size of 1 mm^2^ there is probably only a few mg of sample being measured, so a crude estimate of the detection limit would be in the range 100–500 μg. This would be matrix dependent and further work would be needed to determine the actual detection limits. The detection limit for infrared spectroscopy is probably similar, as the ATR technique used in Figure 4 interrogates a similar sample volume to that of the FT-Raman system.

## Supplementary Material



## Notes

Figure S1. The ^1^H NMR spectrum of PLP-IPAm in DMSO-d6; Figure S2. The ^13^C NMR spectrum of PLP-IPAm in DMSO-d6; Figure S3. The ^31^P NMR spectrum of PLP-IPAm in DMSO-d6; Figure S4. The ^1^H NMR spectrum of PLP-PEA in DMSO-d6; Figure S5. The ^13^C NMR spectrum of PLP-PEA in DMSO-d6; Figure S6. The ^31^P NMR spectrum of PLP-PEA in DMSO-d6; Figure S7. The INS spectrum of PLP-IPAm (4000–50 cm^–1^); Figure S8. The INS spectrum of PLP-PEA (4000–50 cm^–1^); Figure S9. Screenshots from the GaussView software depicting simulated stretching modes within PLP-IPam and PLP-PEA at their predicted wavenumbers; Figure S10. Vibrational modes of a monosubstituted benzene in C2v symmetry, computed using DFT (B3LYP/aug-cc-pVDZ) and labeled according to Gardner and Wright notation; Table S1. The approximate wavenumber ranges for the MiX vibrations; Table S2. The vibrational assignments of PLP-IPAm in the range 4000–400 cm^–1^; Table S3. The vibrational assignments of PLP-PEA in the range 4000–400 cm^–1^; Figure S11. The PXRD pattern collected for synthesized PLP-IPAm (a) and PLP-PEA (b); Figure S12. The collected PXRD pattern of PLP-PEA after attempted recrystallization in N,N-dimethylformamide via vapor diffusion; Figure S13. Two computational models of PLP-IPAm generated using GaussView to investigate the absence of the ν­(C=N) mode in the ATR-IR and FT-Raman spectra (PDF)

## Notes

R.S.D.: Methodology, Validation, Formal analysis, Investigation, Data Curation, Writing—Original Draft, Writing—Review & Editing, Visualization; S.F.P.: Methodology, Validation, Formal analysis, Investigation, Data Curation, Writing—Review & Editing, Visualization, Supervision, Project administration; P.C.: Conceptualization, Resources, Writing—Review & Editing, Supervision, Project administration, Funding acquisition; A.P.: Conceptualization, Writing—Review & Editing; A.P.E.Y.: Resources, Writing—Review & Editing, Supervision, Project administration, Funding acquisition; D.G.: Methodology, Investigation, Writing—Review & Editing; S.T.: Methodology, Writing—Review & Editing; T.J.: Methodology, Resources, Writing—Review & Editing, Visualization, Supervision, Project administration; T. Hyde: Resources, Writing—Review & Editing, Visualization, Supervision, Project administration; A.S.: Methodology, Investigation, Resources; L.W.: Methodology, Investigation; D.L.: Methodology, Validation, Formal analysis, Investigation, Resources, Writing—Review & Editing, Visualization, Supervision, Project administration
