# Supplementary material for: A Vibrational Analysis of Pyridoxal 5′-Phosphate Derivatives: Pyridoxal 5′-Phosphate-isopropylamine and Pyridoxal 5′-Phosphate‑(S)‑1-phenylethylamine
Source: J Phys Chem B. 2025 Dec 29;130(1):33–45. doi: 10.1021/acs.jpcb.5c04584 (PMC12794160; doi:10.1021/acs.jpcb.5c04584)
Supplement: Supplementary file 1 [file jp5c04584_si_001.pdf]

Supporting Information

A Vibrational Analysis of Pyridoxal 5'-Phosphate Derivatives: Pyridoxal 5'-phosphate-isopropylamine and Pyridoxal 5'-phosphate-(*S*)-1-phenylethylamine

Ramandeep S. Dosanjh[a], Stewart F. Parker[a,b], Paul Collier[c], Ahir Pushpanath[d], Andrew P. E. York[c], Damian Grainger[d], Sanita B. Tailor[d], Timothy Johnson[c], Timothy Hyde[c], Lachlan J.N. Waddell[a], Andrew Sutherland[a], and David Lennon[a*]

a School of Chemistry, Joseph Black Building, University of Glasgow, Glasgow, G12 8QQ, UK;
b ISIS Facility, Rutherford Appleton Laboratory Harwell Campus, Chilton, OX11 0QX, UK;
c Johnson Matthey Technology Centre, Blounts Court Road, Sonning Common, Reading, RG4 9NH, UK;
d Johnson Matthey Biocatalysis, 28 Cambridge Science Park, Milton Road, Cambridge, CB4 0FP, UK.

Figure S1. The ^{1}H NMR spectrum of PLP-IPAm in DMSO-*d_6*.

Figure S2. The ^{13}C NMR spectrum of PLP-IPAm in DMSO-*d_6*.

Figure S3. The ^{31}P NMR spectrum of PLP-IPAm in DMSO-*d_6*.

Figure S4. The ^{1}H NMR spectrum of PLP-PEA in DMSO-*d_6*.

Figure S5. The ^{13}C NMR spectrum of PLP-PEA in DMSO-*d_6*.

Figure S6. The ^{31}P NMR spectrum of PLP-PEA in DMSO-*d_6*.

Figure S7. The INS spectrum of PLP-IPAm (4000-50 cm^{-1}).

Figure S8. The INS spectrum of PLP-PEA from (4000-50 cm^{-1}).

Figure S9 Screenshots from the GaussView software depicting simulated stretching modes within PLP-IPam and PLP-PEA at their predicted wavenumbers.

Figure S10. Vibrational modes of a mono-substituted benzene in C_{2v} symmetry, computed using DFT (B3LYP/aug-cc-pVDZ) and labelled according to Gardner and Wright notation.

Table S1. The approximate wavenumber ranges for the $\mathcal{M}_i$X vibrations.

Table S2. The vibrational assignments of PLP-IPAm in the range 4000-400 cm^{-1}.

Table S3. The vibrational assignments of PLP-PEA in the range 4000-400 cm^{-1}.

Figure S11. The PXRD pattern collected for synthesised PLP-IPAm (a) and PLP-PEA (b).

Figure S12. The collected PXRD pattern of PLP-PEA after attempted recrystallisation in *N,N*-dimethylformamide via vapor diffusion.

Figure S13 Two computational models of PLP-IPAm generated using GaussView to investigate the absence of the ν(C=N) mode in the ATR-IR and FT-Raman spectra.

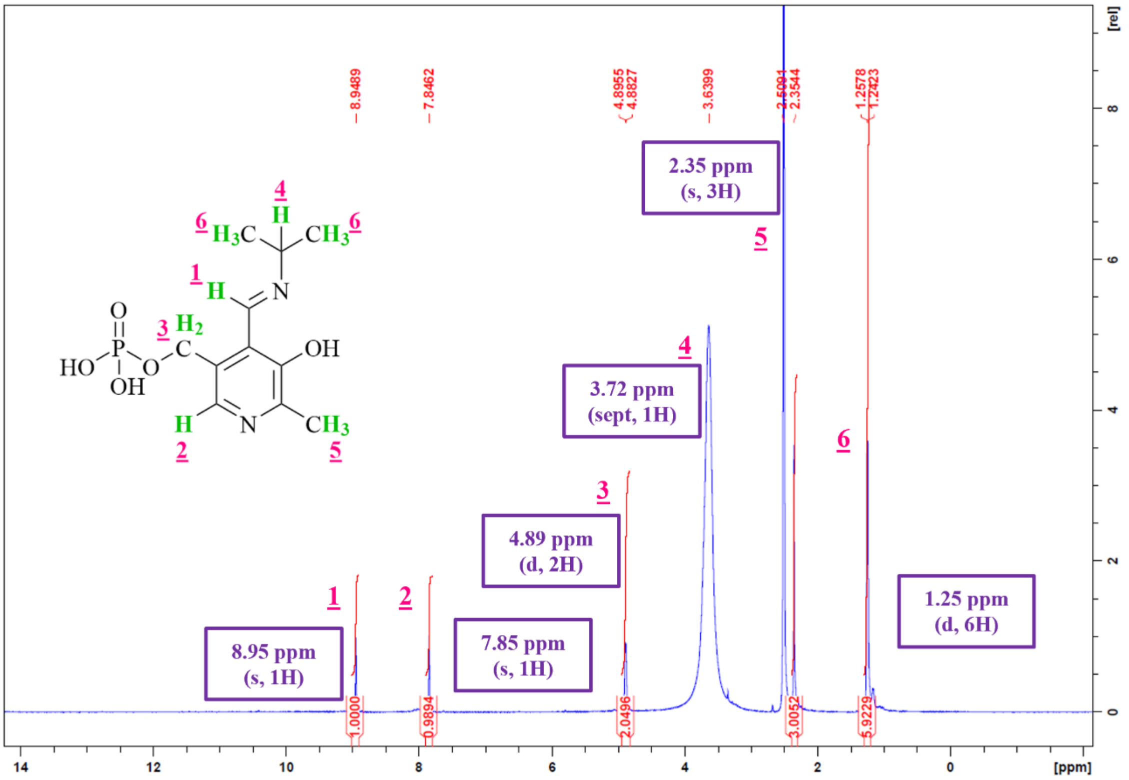

Figure S1. The ^{1}H NMR spectrum of PLP-IPAm in DMSO-d_6.

[^{1}H NMR (400 MHz, DMSO-d_6) δ 8.95 (s, 1H), 7.85 (s, 1H), 4.89 (d, J = 5.1 Hz, 2H), 3.72 (sept, J = 6.2 Hz, 1H), 2.35 (s, 3H), 1.25 (d, J = 6.2 Hz, 6H).]

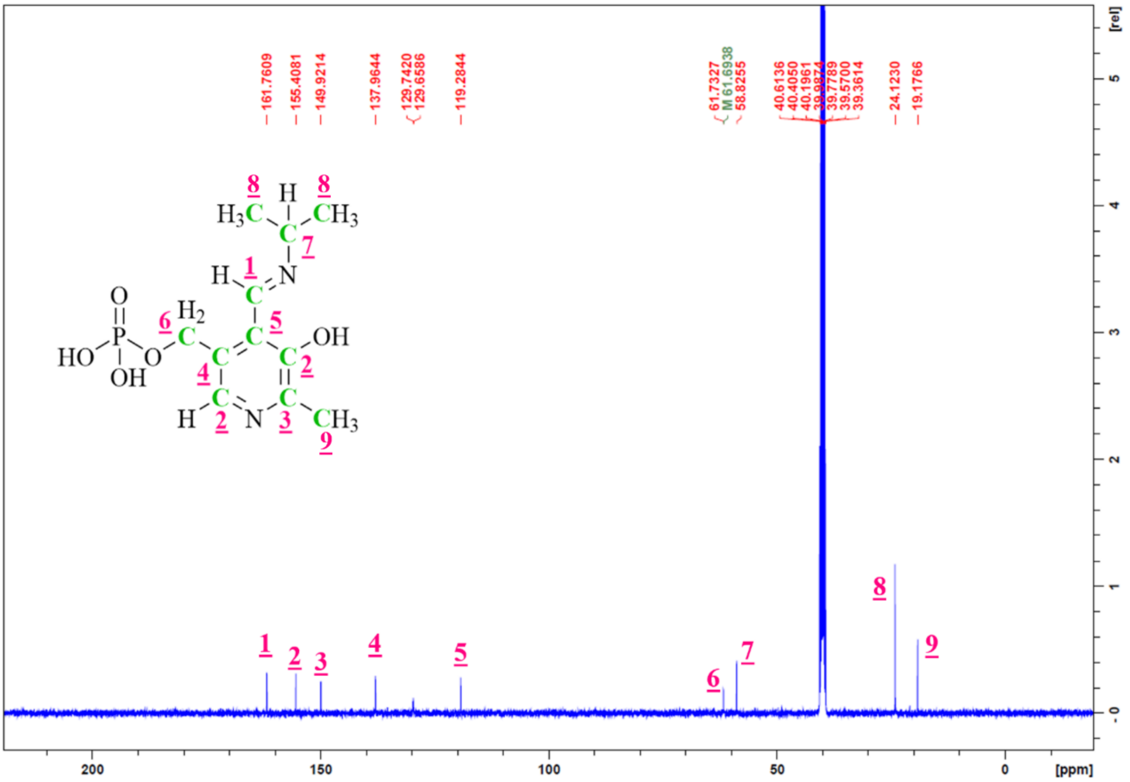

Figure S2. The ^{13}C NMR spectrum of PLP-IPAm in DMSO-*d*$_6$.

[^{13}C NMR (100 MHz, DMSO-*d*$_6$) d 161.8, 155.4, 149.9, 138.0, 129.7, 119.3, 61.7 ($J_{C,P}$ = 3.9 Hz), 24.1, 19.2.]

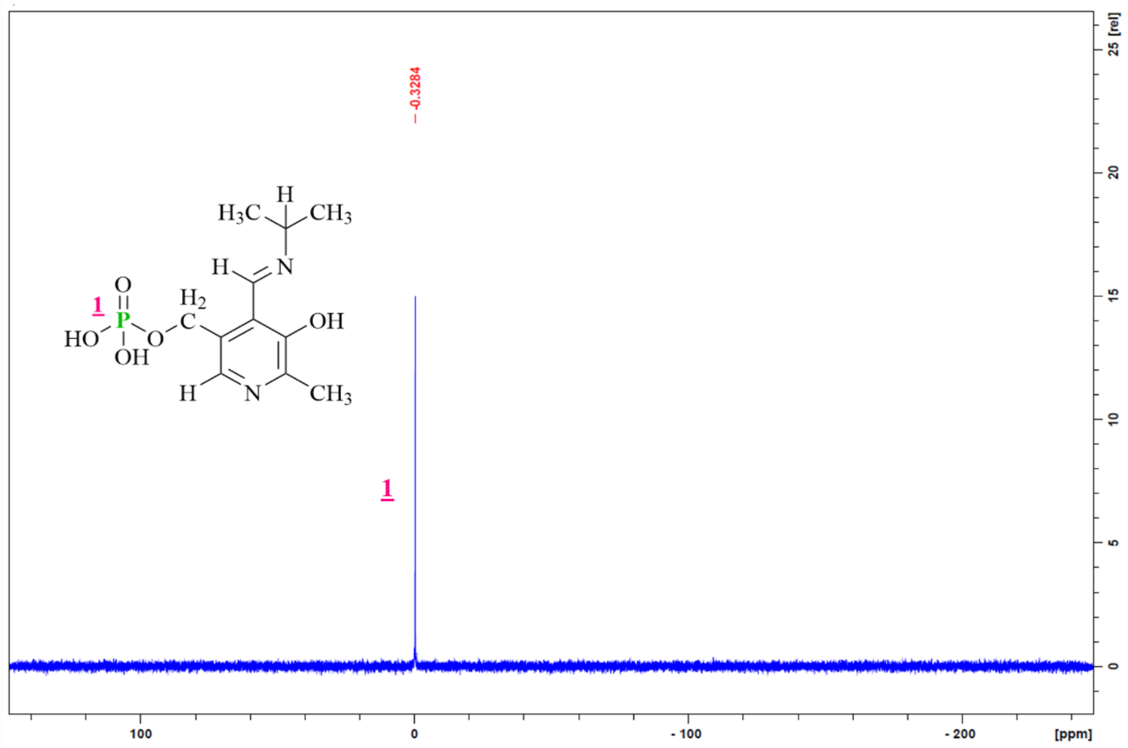

Figure S3. The ^{31}P NMR spectrum of PLP-IPAm in DMSO-d_6.

[^{31}P NMR (162 MHz, DMSO-d_6) δ -0.33.]

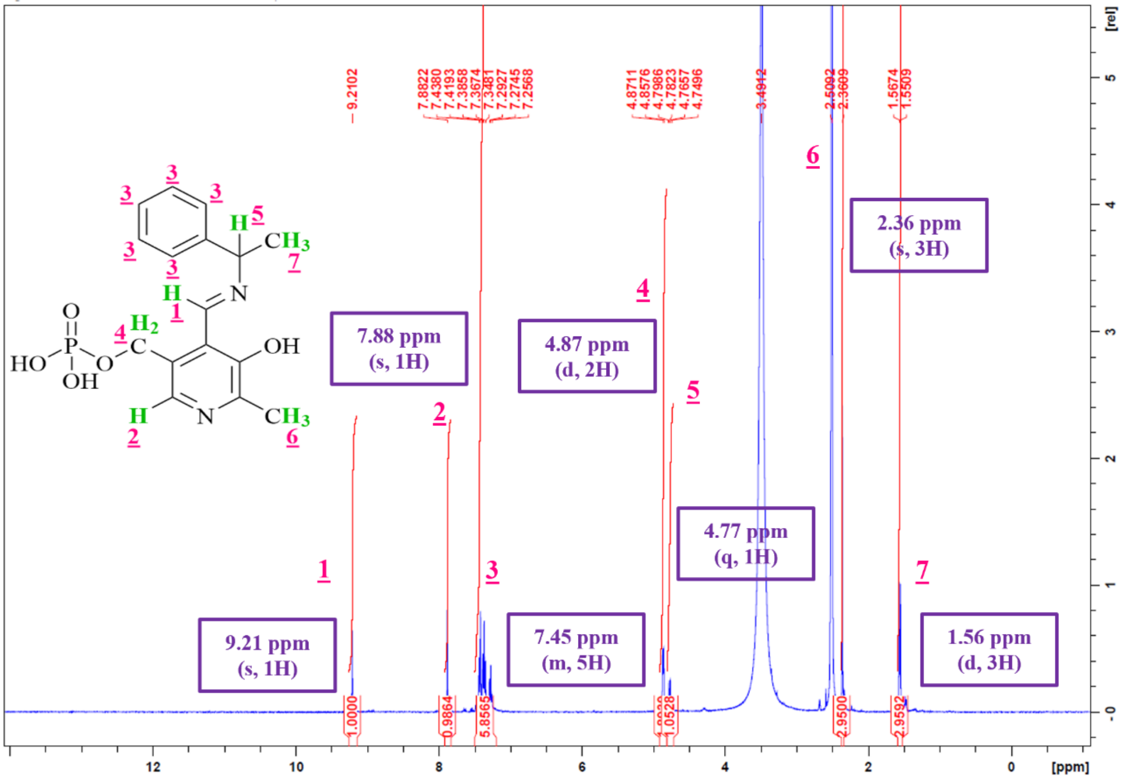

Figure S4. The ^{1}H NMR spectrum of PLP-PEA in DMSO-d_6.

[^{1}H NMR (400 MHz, DMSO-d_6) δ 9.21 (s, 1H), 7.88 (s, 1H), 7.45 (m, 5H), 4.87 (d, J = 5.4 Hz, 2H), 4.77 (q, J = 6.6 Hz, 1H), 2.36 (s, 3H), 1.56 (d, J = 6.6 Hz, 3H).]

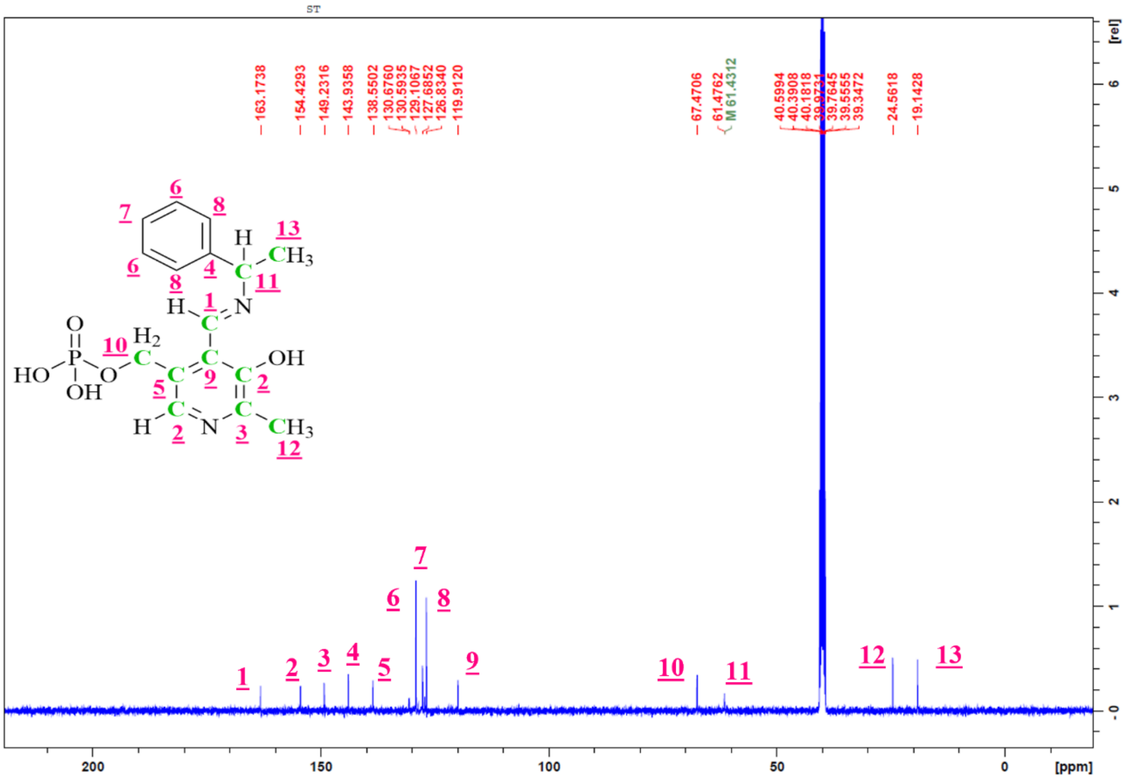

Figure S5. The ^{13}C NMR spectrum of PLP-PEA in DMSO-*d₆*.

[^{13}C NMR (100 MHz, DMSO-d_6) δ 163.2, 154.4, 149.2, 143.9, 138.6, 130.6 ($J_{C,N}$ = 8.3 Hz), 129.1, 127.7, 126.8, 119.9, 67.5, 61.4 ($J_{C,P}$ = 4.5 Hz), 24.6, 19.1.]

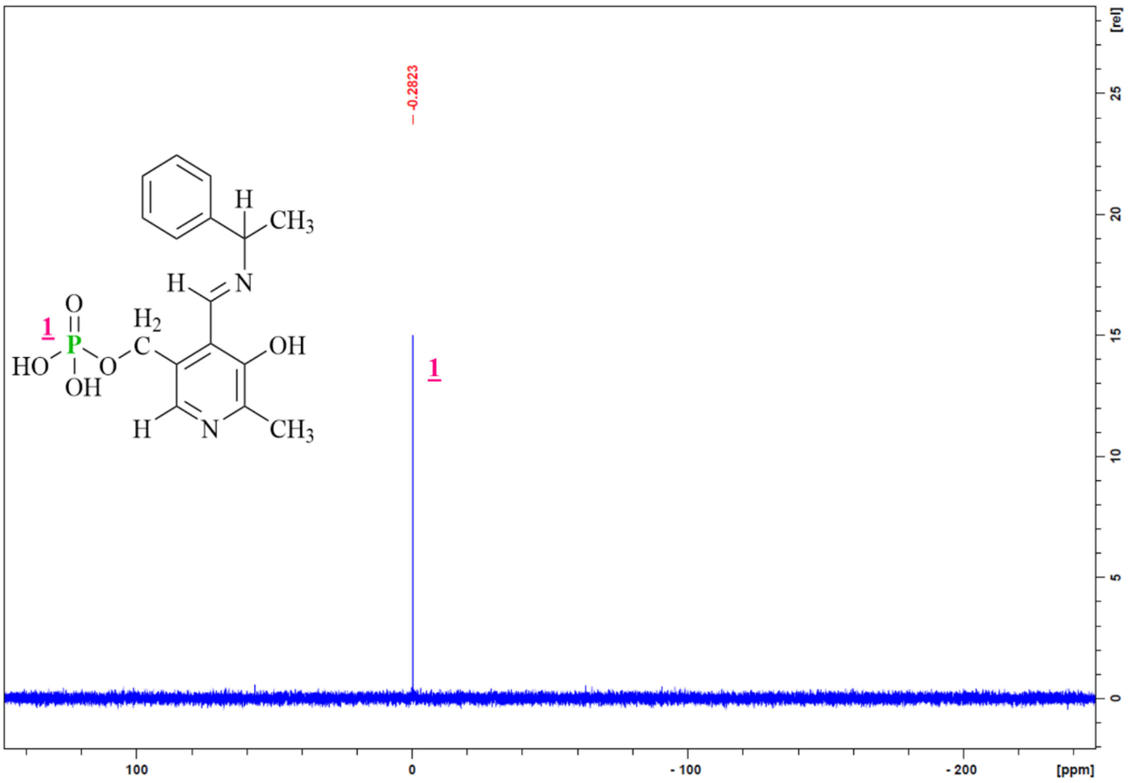

Figure S6. The ^{31}P NMR spectrum of PLP-PEA in DMSO-*d*$_6$.

[^{31}P NMR (162 MHz, DMSO-*d*$_6$) δ -0.28.]

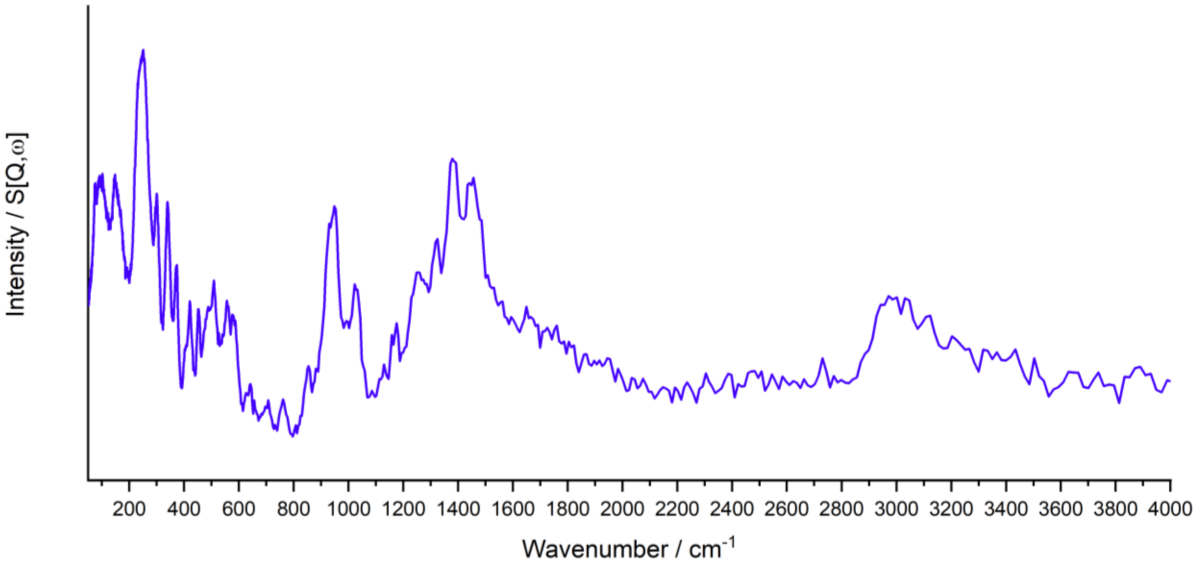

Figure S7. The INS spectrum of PLP-IPAm (4000-50 cm^{-1}).

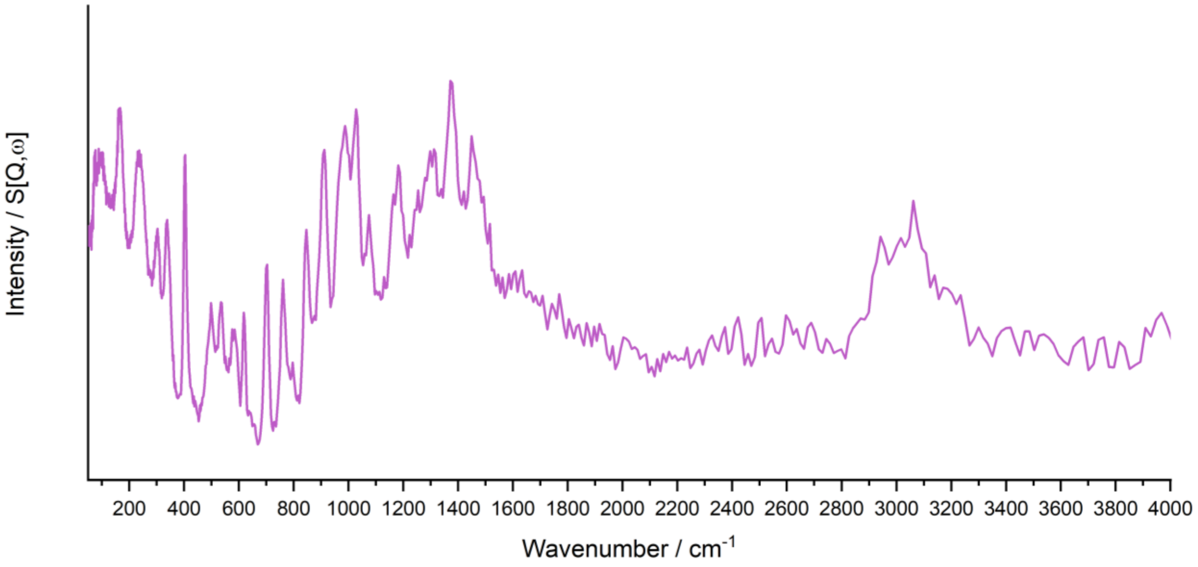

Figure S8. The INS spectrum of PLP-PEA from (4000-50 cm^{-1}).

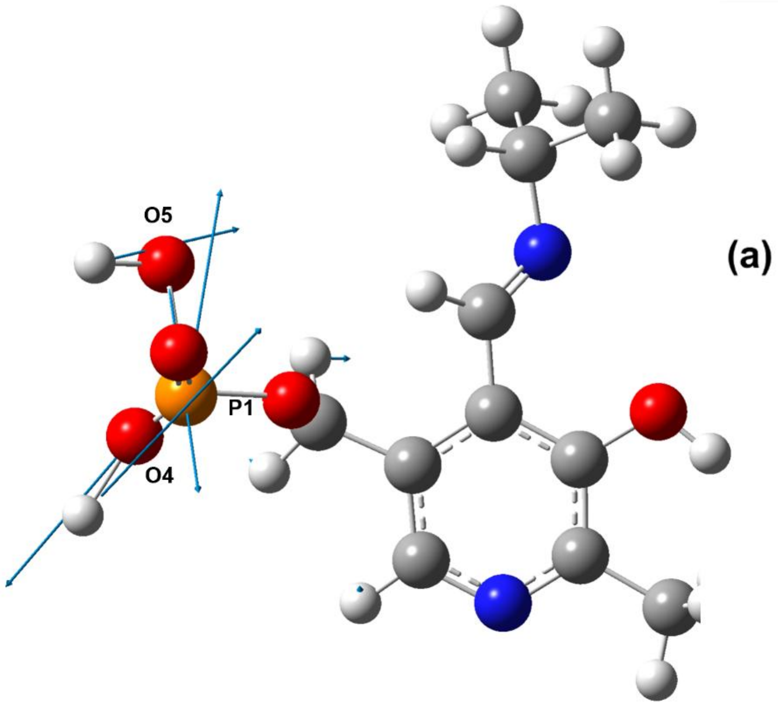

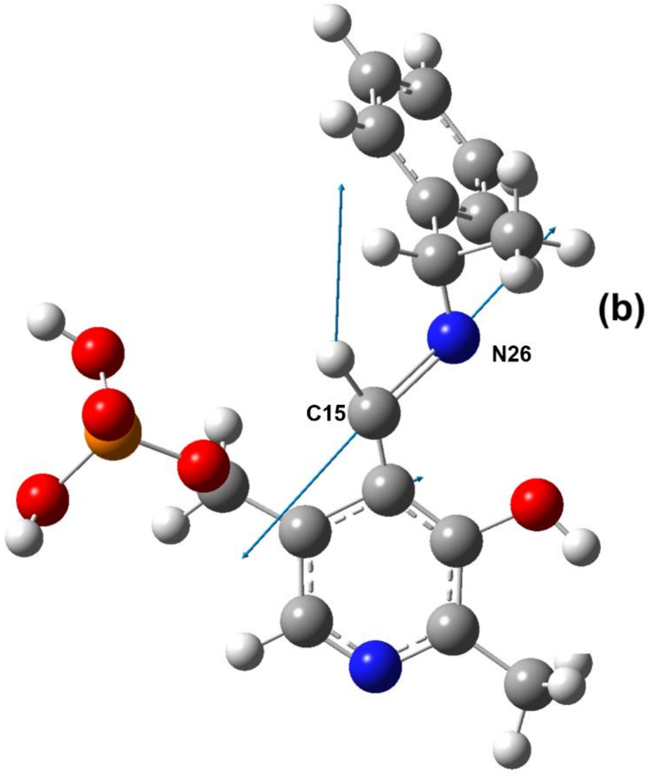

Figure S9 Screenshots from the GaussView software depicting simulated stretching modes within PLP-IPam and PLP-PEA at their predicted wavenumbers: (a) The ν_{as}(O4-P1-O5) mode in PLP-IPAm at 876 cm^{-1} and (b) The ν(C15=N26) mode in PLP-PEA at 1715 cm^{-1} Atoms involved in each vibrational mode are labelled, and blue arrows indicate atomic displacement vectors for clarity.

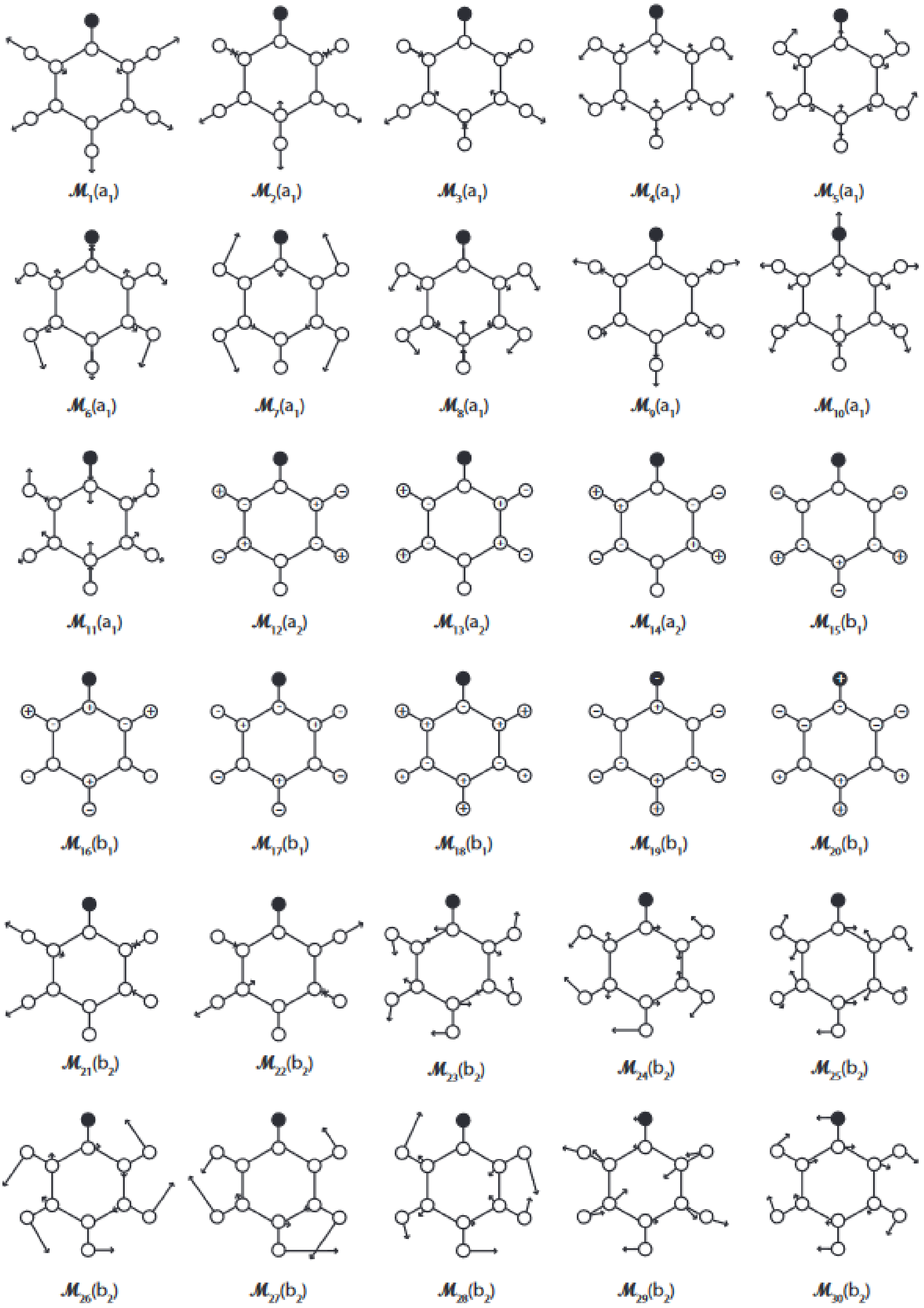

Figure S10. Vibrational modes of a monosubstituted benzene with their associated symmetry designations, computed using DFT (B3LYP/aug-cc-pVDZ) and labelled according to Gardner and Wright notation.[1] Reproduced with permission from the Journal of Chemical Physics.

Table S1. The approximate wavenumber ranges for the $\mathcal{M}_iX$ vibrations according to Gardner and Wright notation.[2] The table is reproduced with permission from the Journal of Chemical Physics.

Mode number (i)	Symmetry (X)	Wavenumber range / cm^{-1}
1	A_1	3100-3060
2	A_1	3090-3040
3	A_1	3060-3000
4	A_1	1610-1570
5	A_1	1510-1460
6	A_1	1300-1050
7	A_1	1180-1140
8	A_1	1040-1010
9	A_1	1010-990
10	A_1	820-640
11	A_1	530-240
12	A_2	980-950
13	A_2	860-810
14	A_2	420-400
15	B_1	1000-960
16	B_1	940-870
17	B_1	800-710
18	B_1	700-670
19	B_1	550 -410
20	B_1	250-160
21	B_2	3100-3030
22	B_2	3070-3020
23	B_2	1590-1570
24	B_2	1470-1410
25	B_2	1340-1310
26	B_2	1300-1250
27	B_2	1180-1120
28	B_2	1090-1060
29	B_2	630-610
30	B_2	410-510

Table S2. The vibrational assignments of PLP-IPAm in the range 4000-400 cm^{-1}. ν = stretch, ν_s = symmetric stretch, ν_{as1} = triple degenerate asymmetric stretch, ν_{as2} = doubly degenerate asymmetric stretch, δ = deformation, δ_s = symmetric deformation, δ_{as} = asymmetric deformation, ρ = rock, τ = twist, ω = wag ipb = in-plane bend, opb = out-of-plane. *vw* = very weak, *w* = weak, *m* = medium, *s* = strong, *vs* = very strong, *sh* = shoulder, *br* = broad.

Approximate description	p-DFT /cm^{-1}	ATR-IR /cm^{-1}	FT-Raman /cm^{-1}	INS /cm^{-1}
δ_s(C29-C27-C33)ipb	405	422 *m*	-	-
δ_s(O2-P1=O6)ipb	439	455 *sh*	-	-
δ_s(O2-P1-O5)ipb	452	455 *sh*	-	-
δ(N27-C27)opb	458	455 *sh*	-	-
δ_s(O2-P1-O4)ipb	466	492 *vs*	502 *w*	508 *m*
δ_{ring}(Pyr)ipb	490	492 *vs*	502 *w*	508 *m*
Out-of-phase δ_s(C9-C8-C13)ipb δ_s(C11-C12-N7)ipb	534	545 *w*	535 *vw*	534 *vw*
δ_{ring}(Pyr)ipb	570	588 *w*	590 *w*	588 *sh*
In-phase δ_s(C8-C9-C11)opb δ_s(C12-N7-C13)opb	578	588 *w*	590 *w*	588 *sh*
Out-of-phase δ_s(C8-C9-C11)opb δ_s(C12-N7-C13)opb	612	634 *s*	-	634 *m*
Out-of-phase δ(C8-C13)opb δ(C11-C12)opb	627	634 *s*	-	634 *m*
δ_{ring}(Pyr)ipb	710	701 *m*	707 *m*	707 *w*
Out-of-phase δ_s(C9-C11-C12)opb δ_s(C8-N7-C13)opb	770	764 *m*	776 *w*	761 *m*
Out-of-phase δ(C9-C8-C13)opb δ(C12-N7-C13)opb	772	764 *m*	776 *w*	761 *m*

In-phase<br>δ(C9-C8-C13)opb<br>δ(C9-C11-C12)opb	805	-	809 *w*	806 *w*
ν(C27-N26)	850	834 *s*	854 *m*	853 *m*
ν_s(O4-P1-O5)	871	834 *s*	854 *m*	853 *m*
ν_{as}(O4-P1-O5)	876	834 *s*	854 *m*	853 *m*
δ(C13-H18)opb	924	916 *vs*	920 *vw*	930 *vs*
In-phase<br>ρ(C29-H_3)<br>ρ(C33-H_3)	936	916 *vs*	950 *m*	948 *vs*
ρ(C14-H_3)	945	916 *vs*	950 *m*	948 *vs*
Mixed modes	952	916 *vs*	950 *m*	948 *vs*
Out-of-phase<br>ρ(C29-H_3)<br>ρ(C33-H_3)	957	916 *vs*	950 *m*	948 *vs*
δ_s(C8-C13-N7)opb	982	984 *sh*	990 *vw*	990 *w*
ν(C10-O2)	993	984 *sh*	990 *v*	990 *w*
δ(C15-H22)opb	996	984 *sh*	990 *v*	990 *w*
Out-of-phase<br>δ(O4-H24)opb<br>δ(O5-H25)ipb	1018	1021 *vs*	1030 *w*	1022 *s*
Mixed mode	1025	1021 *vs*	1030 *w*	1022 *s*
In-phase<br>δ(O4-H24)opb<br>δ(O5-H25)ipb	1035	1021 *vs*	1030 *w*	1022 *s*
ρ(C14-H_3)	1052	1051 *sh*	1057 *vw*	1058 *sh*
ν(C8-C10)	1086	1077 *sh*	1082 *m*	1085 *w*
δ_{as}(C15-N26-C27)opb	1131	1155 *s*	1122 *m*	1130 *w*
ν(N7-C13)	1170	1155 *s*	1170 *w*	1157 *m*
δ_s(C29-C27-C33)opb	1189	1192 *sh*	1170 *w*	1176 *m*
Out-of-phase<br>ν(C8-C13)<br>ν(C11-C12)	1245	1244 *sh*	1244 *m*	1254 *m*
Out-of-phase	1256	1244 *sh*	1244 *m*	1254 *m*

δ_s(C9-C11-C12)ipb<br>δ_s(C8-C13-N7)ipb				
ν(N7-C13)	1269	1263 *sh*	1260 *vw*	1254 *m*
δ_s(C8-C9-C11)ipb	1276	1263 *sh*	1260 *vw*	1254 *m*
ν(P1=O6)	1307	1293 *vw*	1299 *m*	1325 *m*
δ(C13-H10)ipb	1312	1293 *vw*	1299 *m*	1325 *m*
Out-of-phase<br>ν(C8-C13)<br>ν(N7-C12)	1337	1322 *vw*	1323 *w*	1325 *m*
δ(C27-H28)opb	1354	-	1336 *vw*	1378 *s*
In-phase<br>δ(C15-H22)ipb<br>δ(C27-H28)ipb	1357	-	1336 *vw*	1378 *s*
Out-of-phase<br>δ_s(C29-H$_3$)<br>δ_s(C33-H$_3$)	1394	1400 *s*	1376 *s*	1378 *s*
δ_s(C14-H$_3$)	1404	1400 *s*	1376 *s*	1378 *s*
Out of-phase<br>δ(C15-H22)ipb<br>δ(C27-H28)ipb	1408	1400 *s*	1376 *s*	1378 *s*
ω(H15-C10-H17)	1409	1400 *s*	1376 *s*	1378 *s*
In-phase<br>δ_s(C29-H$_3$)<br>δ_s(C33-H$_3$)	1414	1400 *s*	1376 *s*	1378 *s*
ν(C11-O3)	1434	1400 *s*	1406 *s*	-
Out-of-phase<br>τ(C29-H$_3$)<br>τ(C33-H$_3$)	1481	1466 *s*	1455 *s*	1456 *m*
δ_{as}(C14-H$_3$)	1482	1466 *s*	1455 *s*	1456 *m*
Out-of-phase<br>δ_{as}(C29-H$_3$)<br>δ_{as}(C33-H$_3$)	1488	1466 *s*	1455 *s*	1456 *m*
τ(C14-H$_3$)	1489	1466 *s*	1455 *s*	1456 *m*
In-phase	1494	1466 *s*	1455 *s*	1456 *m*

τ(C29-H_3)<br>τ(C33-H_3)				
In-phase<br>δ_{as}(C29-H_3)<br>δ_{as}(C33-H_3)	1510	1466 *s*	1455 *s*	1456 *m*
δ_s(C9-C8-C13)ipb	1515	1466 *s*	1455 *s*	1456 *m*
δ_s(H15-C10-H17)ipb	1517	1466 *s*	1455 *s*	1456 *m*
In-phase<br>ν(C8-C13)<br>ν(N7-C12)	1589	–	1554 *m*	–
In-phase<br>ν(C9-C11)<br>ν(N7-C13)	1632	1628 *s*	1627 *vs*	–
ν(C15=N26)	1713	-	-	-
ν(C27-H28)	2935	2873 *vw*	2873 *sh*	-
ν_s(C14-H_3)	3002	2925 *vw*	2925 *vs*	-
ν(C15-H22)	3007	2925 *vw*	2925 *vs*	-
Out-of-phase<br>ν_s(C29-H_3)<br>ν_s(C33-H_3)	3025	2925 *vw*	2925 *vs*	-
In-phase<br>ν_s(C29-H_3)<br>ν_s(C33-H_3)	3029	2925 *vw*	2925 *vs*	-
ν_{as2}(C14-H_3)	3047	2925 *vw*	2925 *vs*	-
ν_s(H15-C10-H17)	3061	2925 *vw*	2925 *vs*	-
Out-of-phase<br>ν_{as1}(C29-H_3)<br>ν_{as1}(C33-H_3)	3093	2971 *m*	2975 *s*	-
In-phase<br>ν_{as1}(C29-H_3)<br>ν_{as1}(C33-H_3)	3096	2971 *m*	2975 *s*	-
Out-of-phase<br>ν_{as2}(C29-H_3)<br>ν_{as2}(C33-H_3)	3099	2971 *m*	2975 *s*	-

In-phase ν_{as2}(C29-H_3) ν_{as2}(C33-H_3)	3102	2971 *m*	2975 *s*	-
ν_{as}(H15-C10-H17)	3110	2971 *m*	2975 *s*	-
ν_{as1}(C14-H_3)	3135	-	2975 *s*	-
ν(C13-H18)	3146	-	2975 *s*	-
ν(O4-H24)	3828	-	-	-
ν(O3-H23)	3830	-	-	-
ν(O5-H25)	3839	-	-	-

Table S3. The vibrational assignments of PLP-PEA in the range 400-4000 cm^{-1}. ν = stretch, ν_s = symmetric stretch, ν_{as} = asymmetric stretch, ν_{as1} = triple degenerate asymmetric stretch, ν_{as2} = doubly degenerate asymmetric stretch, δ = deformation, δ_s = symmetric deformation, δ_{as} = asymmetric deformation, ρ = rock, τ = twist, ω = wag ipb = in-plane bend, opb = out-of-plane. *vw* = very weak, *w* = weak, *m* = medium, *s* = strong, *vs* = very strong, *sh* = shoulder, *br* = broad.

Approximate description	p-DFT / cm^{-1}	ATR-IR / cm^{-1}	FT-Raman / cm^{-1}	INS / cm^{-1}
$\mathcal{M}_{14}(a_2)$	414	405 *vw*	406 *vw*	404 *vs*
δ_s(O2-P1=O6)ipb	444	497 *s*	502 *m*	-
δ_s(O2-P1-O5)ipb	452	497 *s*	502 *m*	-
δ_s(O2-P1-O4)ipb	467	497 *s*	502 *m*	-
ρ_{ring}(Pyr)	490	497 *s*	502 *m*	500 *m*
Out-of-phase δ_s(C9-C8-C13)ipb δ_s(C11-C12-N7)ipb	531	532 *sh*	531 *w*	536 *m*
$\mathcal{M}_{19}$(b1)	545	556 *sh*	550 *w*	536 *m*
In-phase δ_s(C8-C9-C11)opb δ_s(C12-N7-C13)opb	551	556 *sh*	550 *w*	536 *m*
Out-of-phase δ(C9-C11)opb δ(N7-C13)opb	581	589 *w*	598 *w*	583 *w*
In-phase δ(C9-C8-C13)opb δ(C11-C12-N7)opb	605	632 *m*	619 *m*	619 *m*
Out-of-phase δ(C8-C13)opb δ(C11-C12)opb	629	632 *m*	619 *m*	619 *m*
$\mathcal{M}_{29}(b_2)$	635	632 *m*	619 *m*	619 *m*
δ(C33-C27-N26)ipb	644	632 *m*	619 *m*	619 *m*
δ_{ring}(Pyr)ipb	712	698 *s*	700 vw	703 s
$\mathcal{M}_{18}(b_1)$	713	698 *s*	700 vw	703 s
Out-of-phase δ(C9-C11-C12)opb	771	761 *s*	758 *m*	763 *s*

δ(C8-C13-N7)opb				
Out-of-phase<br>δ(C9-C8-C13)opb<br>δ(C12-N7-C13)opb	771	761 *s*	758 *m*	763 *s*
$\mathcal{M}_{17}(b_1)$	777	761 *s*	758 *m*	763 *s*
$\mathcal{M}_{10}(a_1)$	800	-	794 *s*	798 *w*
In-phase<br>δ(C8-C9-N7)opb<br>δ(C8-C9-C11)opb	808	-	794 *s*	798 *w*
$\mathcal{M}_{13}(a_2)$	858	836 *s*	845 *m*	847 *s*
ν_s(O4-P1-O5)	871	836 *s*	845 *m*	847 *s*
ν_{as}(O4-P1-O5)	876	836 *s*	845 *m*	847 *s*
ν_s(N26-C27-C29)	911	916 *vs*	908 w	911
δ(C13-H18)opb	924	916 *vs*	908 w	911
$\mathcal{M}_{16}(b_1)$	929	916 *vs*	908 w	911
ρ(C14-H_3)	950	916 *vs*	1002 *vs*	986 *br*
ρ(C29-H_3)	978	916 *vs*	1002 *vs*	986 *br*
$\mathcal{M}_{12}(a_2)$	986	986 *sh*	1002 *vs*	986 *br*
Mixed mode	988	986 *sh*	1002 *vs*	986 *br*
ν(C10-O2)	993	986 *sh*	1002 *vs*	986 *br*
$\mathcal{M}_{15}(b_1)$	1001	1028 *vs*	1002 *vs*	986 *br*
$\mathcal{M}_{9}(a_1)$	1015	1028 *vs*	1002 *vs*	986 *br*
In-phase<br>δ(O4-H24)opb<br>δ(O5-H25)ipb	1017	1028 *vs*	1030 *m*	1027 *s*
Mixed mode	1024	1028 *vs*	1030 *m*	1027 *s*
Out-of-phase<br>δ(C15-H22)opb<br>δ(C27-H28)opb	1031	1028 *vs*	1030 *m*	1027 *s*
Out-of-phase<br>δ(O4-H24)opb<br>δ(O5-H25)ipb	1034	1028 *vs*	1030 *m*	1027 *s*
$\mathcal{M}_{8}(a_1)$	1049	1028 *vs*	1030 *m*	1027 *s*
ρ(C14-H_3)	1052	1075 *s*	1075 *br*	1027 *s*

ν(C8-C10)	1081	1075 *s*	1075 *br*	1075 *s*
ν(C27-C29)	1089	1075 *s*	1075 *br*	1075 *s*
$\mathcal{M}_{28}(b_2)$	1100	1125 *w*	1075 *br*	1075
ν(N26-C27)	1142	1125 *w*	-	
$\mathcal{M}_{27}(b_2)$	1181	1180 *br*	1184 *m*	1182 *s*
$\mathcal{M}_{7}(a_1)$	1199	1180 *br*	1205 *w*	1182 *s*
$\mathcal{M}_{6}(a_1)$	1215	1213 *br*	1215 *w*	1253 *m*
Out-of-phase<br>ν(C8-C13)<br>ν(C11-C12)	1245	1246 *vw*	1245 *m*	1253 *m*
Out-of-phase<br>δ(C9-C11-C12)ipb<br>δ(C8-C13-N7)ipb	1256	1246 *vw*	1245 *m*	1253 *m*
ν_{as}(C8-C9-C15)	1269	1264 *s*	1262 *sh*	1253 *m*
δ_s(C8-C9-C11)ipb	1276	1264 *s*	1262 *sh*	1253 *m*
$\mathcal{M}_{26}(b_1)$	1305	1297 *m*	–	1316 *m*
ν(P1=O6)	1308	1297 *m*	1296 s	1316 *m*
δ(C13-H10)ipb	1313	1297 *m*	1296 s	1316 *m*
Out-of-phase<br>ν(C8-C13)<br>ν(N7-C12)	1338	-	-	1316 *m*
In-phase<br>δ(C15-H22)ipb<br>δ(C27-H28)ipb	1340	-	-	1316 *m*
$\mathcal{M}_{25}(b_2)$	1348	1360 vw	1375 *s*	1378 *s*
$\mathcal{M}_{26}(b_2)$	1375	1375 *sh*	1375 *s*	1378 *s*
δ_s(C29-H_3)	1400	1401 *s*	1375 *s*	1378 *s*
δ_s(C14-H_3)	1404	1401 *s*	1375 *s*	1378 *s*
ω(H15-C10-H17)	1409	1401 *s*	1404 *s*	1378 *s*
Out of-phase<br>δ(C15-H22)ipb<br>δ(C27-H28)ipb	1413	1401 *s*	1404 *s*	1378 *s*
ν(C11-O3)	1434	1449 *s*	1450 *m*	1420 *w*
$\mathcal{M}_{24}(b_2)$	1482	1493 *m*	1482 *vw*	1450 *s*

δ_{as}(C14-H_3)	1482	1493 *m*	1482 *vw*	1450 *s*
τ(C29-H_3)	1487	1493 *m*	1482 *vw*	1450 *s*
τ(C14-H_3)	1490	1493 *m*	1482 *vw*	1450 *s*
δ_{as}(C29-H_3)	1496	1493 *m*	1482 *vw*	1450 *s*
ν(C8-C9)	1515	–	1448	–
δ_s(H15-C10-H17)	1518	–	1448	–
$\mathcal{M}_5(a_1)$	1524	1549 *br*	1553 *m*	–
In-phase<br>ν(C8-C13)<br>ν(N7-C12)	1590	1549 *br*	1553 *m*	–
$\mathcal{M}_{23}(b_2)$	1623	1627 *s*	1626 *vs*	–
In-phase<br>ν(C9-C11)<br>ν(N7-C13)	1632	1627 *s*	1626 *vs*	–
$\mathcal{M}_4(a_1)$	1642	1627 *s*	1626 *vs*	–
ν(C15=N26)	1715	1688 *m*	1688 *m*	–
ν(C27-H28)	2949	–	2884 *sh*	–
ν_s(C14-H_3)	3002	–	2928 *s*	–
ν(C15-H22)	3013	–	2928 *s*	–
ν_s(C29-H_3)	3031	–	2928 *s*	–
ν_{as2}(C14-H_3)	3047	–	2928 *s*	–
ν_s(H15-C10-H17)	3061	–	2928 *s*	–
ν_{as2}(C29-H_3)	3101	–	2979 *m*	–
ν_{as}(H15-C10-H17)	3110	–	2979 *m*	–
ν_{as1}(C29-H_3)	3110	–	2979 *m*	–
ν_{as1}(C14-H_3)	3135	–	2979 *m*	–
ν(C13-H)	3147	–	2979 *m*	–
$\mathcal{M}_3(a_1)$	3151	–	3062 *s*	–
$\mathcal{M}_{22}(b_2)$	3160	–	3062 *s*	–
$\mathcal{M}_2(a_1)$	3171	–	3062 *s*	–
$\mathcal{M}_{21}(b_2)$	3183	–	3062 *s*	–
$\mathcal{M}_1(a_1)$	3192	–	3062 *s*	–
ν(O4-H24)	3828	–	–	–

ν(O3-H23)	3829	–	–	–
ν(O5-H25)	3839	–	–	–

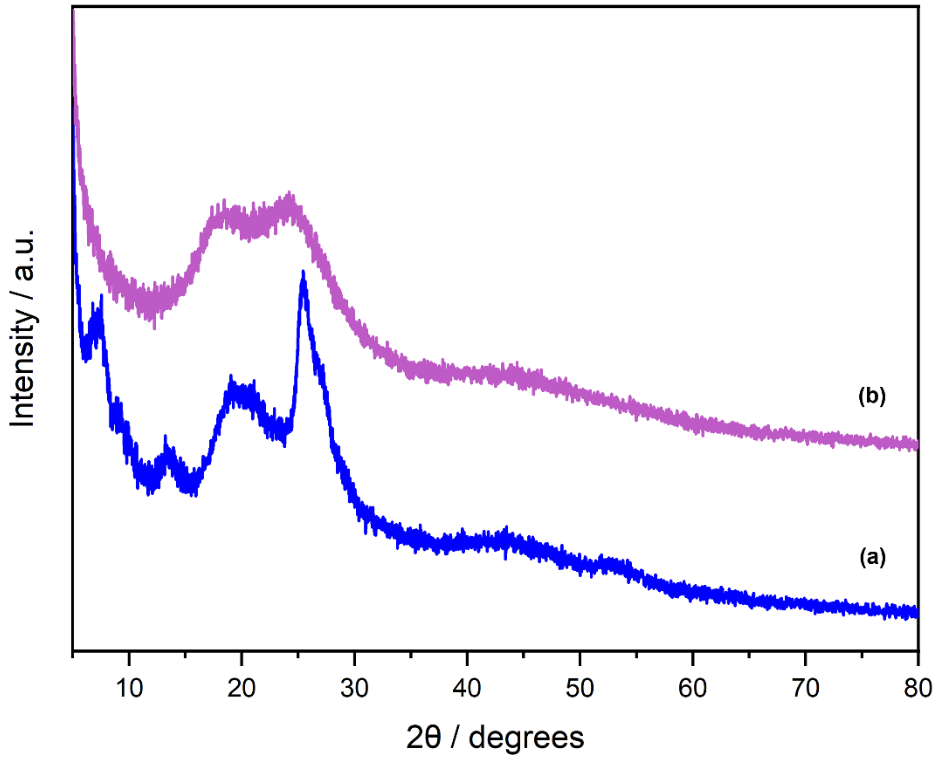

Figure S11. The PXRD pattern collected for synthesised PLP-IPAm (a) and PLP-PEA (b).

Attempted recrystallisation of aldimines 1 and 2 by vapor diffusion

PLP-IPAm or PLP-PEA (0.020 g) was dissolved in dimethyl sulfoxide or *N,N*-dimethylformamide (0.4 mL) in a vial and placed in a larger vial charged with ethyl acetate, diethyl ether, tetrahydrofuran or dichloromethane (5 mL). The experiment was allowed to stand in the dark at either 5 °C or 20 °C for 24–168 h. Any solid recovered was analysed under a microscope, under all conditions samples appeared amorphous.

Attempted recrystallisation of aldimines 1 and 2 by solvent diffusion

PLP-IPAm or PLP-PEA (0.020 g) was dissolved in dimethyl sulfoxide or *N,N*-dimethylformamide (0.4 mL) in a vial and ethyl acetate or diethyl ether 0.6 mL) was carefully layered on top. The experiment was allowed to stand in the dark at either 5 °C or 20 °C for 72 h. Any solid recovered was analysed under a microscope, under all conditions samples appeared amorphous.

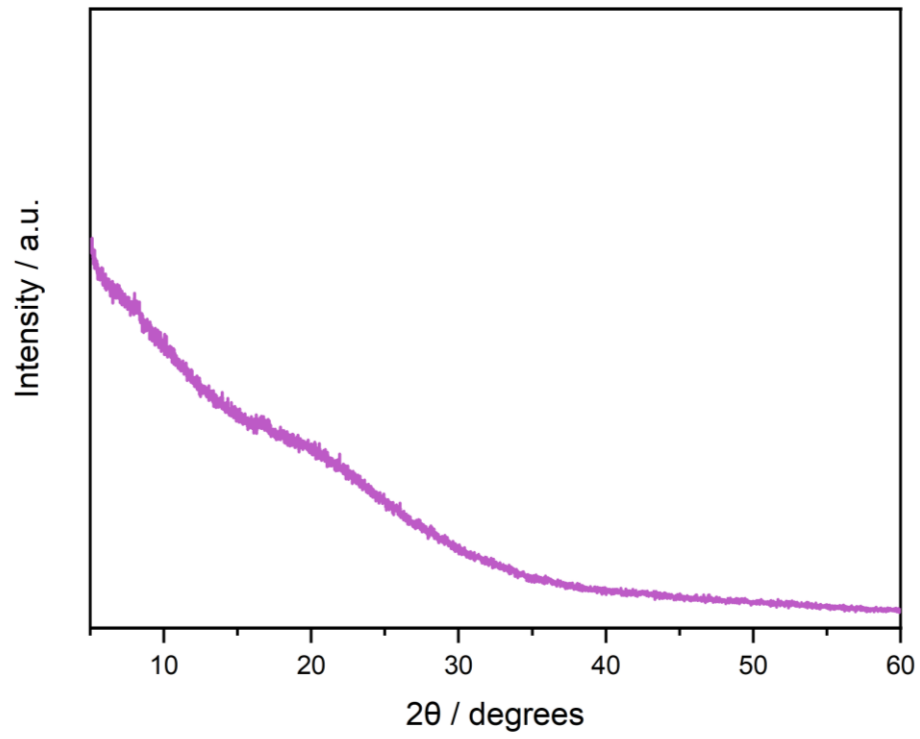

Figure S12. The collected PXRD pattern of PLP-PEA after attempted recrystallisation in *N,N*-dimethylformamide via vapor diffusion.

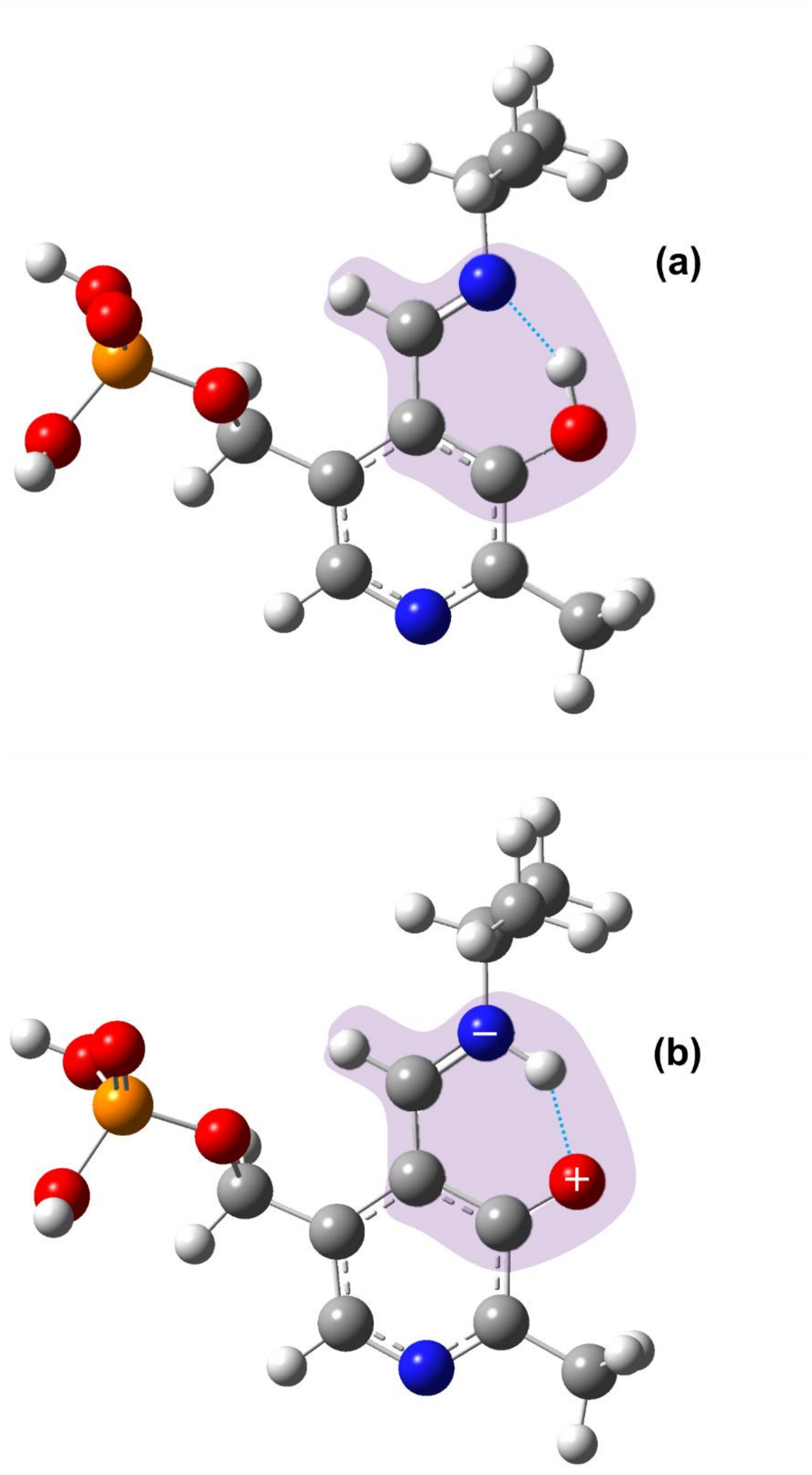

Figure S13 Models of PLP-IPAm generated using GaussView for uncharged (a) and charged (b) configurations featuring an internal hydrogen bond stabilising a six-membered ring

Reference

(1) Gardner, A. M.; Wright, T. G. Consistent Assignment of the Vibrations of Monosubstituted Benzenes. *J. Chem. Phys.* **2011**, *135* (11), 114305. https://doi.org/10.1063/1.3638266.
